# Supplementary material for: Acute stress during witnessing injustice shifts third-party interventions from punishing the perpetrator to helping the victim
Source: PLoS Biol. 2024 May 16;22(5):e3002195. doi: 10.1371/journal.pbio.3002195 (PMC11098560; doi:10.1371/journal.pbio.3002195)
Supplement: S1 Text — (DOCX) [file pbio.3002195.s001.docx]

Supplementary Materials for

**Acute stress reshapes third-party punishment and help decisions: Behavioral evidence and neurocomputational mechanisms.**

Example: Huagen Wang *et al.*

*Corresponding author. Email: E-mail: [liuchao@bnu.edu.cn](mailto:liuchao@bnu.edu.cn)

Supplementary Text

Participants

Fifty-three right-handed **male** volunteers were recruited and randomly assigned to either the stress condition (CPT) or the control condition; Data of one participant was removed from analyses, as he failed to understand the task. The final sample included 52 participants (stress: n = 27, mean age = 23.27 years, *SD* = 2.85 years; control: n = 25, mean age = 23.68 years, *SD* = 2.48 years). (1). In a structured phone interview prior to the experiment, participants who met the following conditions were excluded included hormonal contraception, prescription or drug consumption, smoking, alcohol abuse, a history of chronic disease or mental condition, and a major evaluation within two weeks. Furthermore, participants were instructed to abstain from physical activity, meals, and caffeine consumption for 2 hours prior to the study. (2). All study procedures were approved by the Institutional Review Board of the State Key Laboratory of Cognitive Neuroscience and Learning at Beijing Normal University. All participants signed written informed consent.

Experimental procedures

Testing sessions took place in the afternoon (1:30–5:30) to control variability in diurnal cortisol secretion. Upon arrival at the lab, participants were guided to the testing room, where they were given a rundown of the experiment and asked to complete questionnaires (psychological and personality tests, see SI) for 20 minutes. Subsequently, the baseline heart rate (HR1) was recorded for 3 minutes, the saliva sample (S1) and the Positive and Negative Affect Scale (PANAS, PA1, and NA1) were measured. Then they were randomly assigned to CPT or control conditions. Heart rate (HR2) was recorded across the whole CPT for 3 minutes. After the CPT or a control manipulation, the saliva sample (S2) and the PANAS (PA2 and NA2) were collected immediately. Then, after 10 minutes’ rest and 10 minutes’ T1 structural image collection in the MRI scanner, the subjects completed the first run of the Third-Party Intervention task (TPI). The third saliva sample (S3) and the PANAS (PA3 and NA3) were measured 25–30 minutes after the CPT (run 1 for 10 minutes). And the participants finished their remaining 2 runs of TPI in the MRI scanner for 20 minutes. The fourth saliva sample (S4) and the PANAS (PA4 and NA4) were measured 50 minutes after the CPT.

**Stress Manipulation**

After the baseline saliva sample was collected, participants were randomly assigned to either a stress or control condition. Stress induction involved a cold pressor task (CPT) wherein participants submerged their left hand to the wrist in 0–4°C ice water for 3 consecutive minutes ^[1]^. If participants failed to complete the CPT, they were excluded from the study. The participants in the control group submerged their left hand in warm water (35–37°C) for 3 consecutive minutes. The CPT has been shown to reliably activate the sympathetic nervous system (SNS) and the hypothalamic-pituitary-adrenal (HPA) axis, as demonstrated by elevations in physiological and endocrine (i.e., cortisol) responses, and it has been used to evoke a stress response.

**Saliva Cortisol Collection, Storage and Analysis**

Saliva samples were obtained from participants at four time points before and after the stress/control manipulation to determine stress-induced changes in cortisol concentrations (Sarstedt, Rom-melsdorf, Germany).

The participants were required to hold a saliva-collecting swab in their mouth for approximately 3 minutes until the saliva completely soaked the swab. Saliva samples were held at -80°C until they were analyzed. To rule out other hormonal effects correlated with prosocial activity, we measured testosterone and oxytocin levels in saliva. The samples were thawed and centrifuged for 5 minutes at 3500 rpm. The concentrations of salivary cortisol were analyzed using electrochemiluminescence immunoassay (Cobas e 601, Roche Diagnostics, Nümbrecht, Germany), and those of testosterone were analyzed using an enzyme-linked immunoassay kit developed for saliva (Salimetrics, State College, PA) with sensitivity of 0.500 nmol/L (lower limit). The standard range in the assay was 0.5–1,750 nmol/L for cortisol. The intra- and inter-assay coefficient variations for cortisol were below 10%, and we did not test the precision of testosterone and oxytocin because there was not enough saliva.

**Heart rate Collection**

Furthermore, as markers of sympathetic nervous system activity, heart rate was continuously monitored in the CPT stage (3 min) with a Polar WearLink + heart rate monitor (POLAR RCX3) to determine the effects of the stress induction versus the control task, and heart rate was also monitored for 3 minutes as a baseline before the CPT.

**Psychological** **measures**

The Positive and Negative Affect Scale (PANAS) was used to measure the subjective affective states of participants at each designated instant. The scale has a total of 20 items describing different feelings and emotions, including 10 items for positive affects (e.g., “interested”, “excited”) and 10 items for negative affect (e.g., “nervous”, “scared”). The participants were asked to score each item on a 5-point scale based on their instant affective state, from 1 (very slightly or not at all) to 5 (extremely). The average scores of positive affect (PA) and negative affect (NA) were calculated. Besides, studies have shown that some personality traits (such as impulsiveness) and empathy ^[2-3]^ also have a very important impact on prosocial behavior, therefore, some personality factors were also considered in this study. Before the experiment, participants completed an online survey (implemented via Qualtrics software, 2009, Provo, UT, USA. https://www.qualtrics.com), which included demographic questions and some personality measures: Perceived Stress Scale (PSS) ^[4]^; State-Trait Inventory for Cognitive and Somatic Anxiety (STICSA) ^[5]^; Social Value Orientation (SVO)^[6]^; Behavioral Inhibition System and Behavioral Activation System Scales (BIS/BAS Scales)^[7]^; Interpersonal Reactivity Index (IRI)^[8]^; Altruistic personality scale (APS)^[9]^.

**Third-party Intervention Task**

This task includes three players. The player A (proposer) received an endowment of 100 MUs per round and could decide how to distribute it between himself/herself and the player B (recipient) in units of 5-MUs (i.e., 0, 5, 10, 15, and 20). The player B had to passively accept the proposal. The player C (the third party) received an endowment of 50 monetary units (MUs, 10 MUs = 1 Chinese yuan) per round and was instructed to observe the collection between player A and player B. In the fMRI scanner, participants believed that they were randomly assigned to player C via a massive drawing process. Participants had three options: transferring MUs to reduce player A’s MUs, transferring MUs to increase player B’s MUs, or keeping all the MUs for themselves. If participants wanted to reduce player A’s money (or increase player B’s money), they needed to determine how many MUs to move from their own 50 MUs and deduct the proposer's Mus in units of 5 MUs. Following that, he can continue to decide how much MUs to move from their remaining MUs to compensate the recipient. The number of trials was pre-programmed to 30 trials for one run, a total of 3 runs. The offers were created specifically (i.e., the average offer ratio was 90/10 80/20, 70/30, 60/40, 50/50, but the actual offers shown to the participants fluctuated between 1% and 2%, e.g., 91/9, 88/12).

There are a few specifics to note: 1) When player C decided to deduct A’s MUs or increase B’s MUs, the cost ratio was 1:3, as previously reported ^[2]^, which means that every MU transferred by player C can be deducted or increased by 3 MUs to player A or player B, respectively. 2) In the expression of instructions, we used “player A, B, and C” instead of “dictator”, “recipient” and “observer” and “deduct” and “increase” in place of “punish” and “help” 3) Player A and B were not real; we had pre-programmed the allocation chosen by player A. 4) For each participant, the order of the trials in each run was randomized.

**Computational modeling procedures.**

In addition to the four models mentioned in the main text, we also tried two other models:

***Model 5: Self and other regarding inequality aversion model:1***

This model assumed that participants made decisions both weighting the payoff inequality between themselves and others versus the violator (who always has more than or equal to 50 tokens) and weighting the

difference between themselves and the other two participants. The participants’ goal was to eliminate inequality aversion by both punishment and help. The utility function was formalized as follows:

$$\begin{aligned} U_{i\left( t \right)}\left( S_{i\left( t \right)}^{p},S_{i\left( t \right)}^{h} | \alpha_{i},\beta_{i},{envy}_{i},{guilt}_{i} \right)= \pi_{i\left( t \right)}-\left\{ \max\left[ \alpha_{i}\cdot\left( x_{1\left( t \right)}-50 \right)-3S_{i\left( t \right)}^{p}, 0 \right]-\min\left[ \beta_{i}\cdot\left( x_{2\left( t \right)}-50 \right)+3S_{i\left( t \right)}^{h},0 \right] \right\} \\ -\mathrm{envy}_{i} \cdot\mathrm{DI}_{i} -\mathrm{guilt}_{i}\cdot\mathrm{AI}_{i} \#\left( AUTONUM \backslash* Arabic \right) \end{aligned}$$

where DI is disadvantageous inequality and AI is advantageous inequality aversion, which is calculated as follows:

$${DI}_{t}=\max\left[ x_{1\left( t \right)}-3S_{i\left( t \right)}^{p}-\pi_{i\left( t \right)},0 \right]+max\left[ x_{2\left( t \right)}+3S_{i\left( t \right)}^{h}-\pi_{i\left( t \right)},0 \right] \left( AUTONUM \backslash* Arabic \right)$$

$${AI}_{t}=\max\left[ \pi_{i\left( t \right)}{-(x}_{1\left( t \right)}-3S_{i\left( t \right)}^{p}),0 \right]+max\left[ {\pi_{i\left( t \right)}-(x}_{2\left( t \right)}+3S_{i\left( t \right)}^{h}),0 \right] \left( AUTONUM \backslash* Arabic \right)$$

Note that in each trial π = 50 – S_p_ - S_h_.

***Model 6: Self and other regarding inequality aversion model:2***

$$\begin{aligned} U_{i\left( t \right)}\left( S_{i\left( t \right)}^{p},S_{i\left( t \right)}^{h} | \alpha_{i},\beta_{i},\lambda_{i} \right)=\lambda_{i} \cdot\pi_{i\left( t \right)}-(1-\lambda_{i})\left\{ \max\left[ \alpha_{i}\cdot\left( x_{1\left( t \right)}-50 \right)-3S_{i\left( t \right)}^{p}, 0 \right]-\min\left[ \beta_{i}\cdot\left( x_{2\left( t \right)}-50 \right)+3S_{i\left( t \right)}^{h},0 \right] \right\} \\ \#\left( AUTONUM \backslash* Arabic \right) \end{aligned}$$

In this model, we incorporate the idea that participants exhibit variations not only in their sensitivity to inequity (represented by alpha and beta terms), but also in the extent to which they prioritize their own costs and gains compared to those of others. To capture this trade-off, we have introduced a parameter λ.

The overview of model comparisons.

| **Model** | **Description of the models** | **AIC** |
| --- | --- | --- |
| Model 1 | Baseline model | 477.807038483339 |
| Model 2 | Self-regarding inequality aversion model | 392.351260533234 |
| Model 3 | Other-regarding inequality aversion model with shared parameters for estimating the severity of punishment and help. | 381.167204053846 |
| Model 4 | Other-regarding inequality aversion model | **361.187649224120** |
| Model 5 | Self-Other-regarding inequality aversion model 1 | 397.381785153329 |
| Model 6 | Self-Other-regarding inequality aversion model 2 | 374.434780052375 |

Functional Magnetic Resonance Imaging (fMRI) Procedure

**Imaging data acquisition and preprocessing**

Brain imaging data were acquired on a 3T Prisma MR scanner (Siemens, Erlangen, Germany) with a 64-channel phased-array head-neck coil for signal reception. During the tasks, blood oxygen level-dependent (BOLD) signals were acquired with a prototype simultaneous multi-slice echo-planar imaging (EPI) sequence (echo time, 30 ms; repetition time, 2000 ms; field of view, 224 mm × 224 mm; matrix, 112 × 112; inplane resolution, 2 mm × 2 mm; flip angle, 90 degree; slice thickness, 2.0 mm; gap, 15%; the number of slices, 62; slice orientation, transversal; bandwidth, 2232 Hz/Pixel; slice acceleration factor, 2). Field map images were acquired using a vendor-provided Siemens gradient echo sequence (gre field mapping: echo time 1, 4.92 ms; echo time 2, 7.38 ms; repetition time, 620 ms; flip angle, 60 degree; bandwidth, 565 Hz/Pixel) with the same geometry and orientation as the EPI image. A high-resolution 3D T1 structural image (3D magnetization-prepared rapid acquisition gradient echo; 0.5 mm × 0.5 mm × 1 mm resolution) was also acquired. Image preprocessing was performed using the Statistical Parametric Mapping package (SPM12, RRID: SCR_007037; Welcome Department of Imaging Neuroscience, London, United Kingdom). EPI volumes were realigned to the first volume, corrected for geometric distortions using the field map, coregistered to the T1 image, normalized to a standard template (Montreal Neurological Institute, MNI), resampled to 2 × 2 × 2 mm^3^ voxel size, and spatially smoothed with an isotropic 8 mm full-width at half-maximum Gaussian kernel.

**fMRI data analysis**

During the conversion process, the first three images at the beginning of the functional runs were discarded to enable the signal to achieve steady-state equilibrium between radio frequency pulsing and relaxation. Images were motion corrected for three translational and three rotational directions.

**General linear model**

We regressed the fMRI time series into three general linear models (GLMs) to investigate how acute stress affected the brain’s decision circuitry. We looked for neural activity associated with inequity on the decision stage, i.e., the degree of inequity between the proposer and recipient (GLM_ Inequity) with the first GLM, and in this model, we also check the stress manipulation effect. In the second GLM (GLM_ Choice), we aimed to recognize brain regions whose behavior was associated with punishment and help choice under the unfair condition. The third GLM (GLM_ Utility) sought to identify brain regions coding utility and severity preference between punishment and help during the transfer stage. For the main contrasts, the individual voxel threshold was set to P < 0.001. We performed whole-brain corrections for multiple comparisons at the cluster level (P FWE < 0.05). Furthermore, since we had an a priori hypothesis that the amygdala was related to the "inequity," we used small volume correction (SVC) based on anatomically defined bilateral amygdala region of interests (ROIs) and corrected P FWE <0.05. The anatomical ROIs of the bilateral amygdala were created using the SPM Wake Forest University (WFU) Pickatlas toolbox (www.ansir.wfubmc.edu, version 3.0).

**In the first model (GLM_ Inequity)**, we computed a GLM with a parametric design to identify brain regions coding “Inequity” on the decision window when participants were informed how many MUs had been distributed by the proposer. In each trial, the “Inequity” δ was defined as “δ = |MU Proposer – MU Recipient|”48. Four regressors were included in the GLM in the following order: (i) onset of the decision stage at the beginning of each trial when participants saw the distributions, (ii) parametric modulation of the trial-wise “Inequity”, (iii) onset of the first transfer stage, and (iv) onset of the second transfer stage. We modeled BOLD responses at these onsets as stick functions. All regressors and six head movement regressors of no interest were convolved with a canonical hemodynamic response function. For each event, onset regressor parameter estimates were obtained and contrast images of each of the parameters against zero were generated. The obtained images were transferred to a second-level random-effects analysis using two-sample t-test and conjunction analysis to compare the stress and control groups.

**In the second model (GLM_ Choice)**, we focused on the decision window when participants responded to the trials of unfair distribution (8:2 & 9:1). We defined the following two onset regressors of interest: (i) onset of the punishment choice of unfair trials, (ii) onset of the help choice of unfair trials. We also defined the following uninterested regressors: (iii) onset of the punishment transfer of unfair trials, (iv) onset of the help transfer of unfair trials, (v) onset of all the choices of relative unfair trials (7:3 & 6:4, for punishment, help and keep choice), (vi) all the transfers of relative unfair trials (7:3 & 6:4, for punishment, help and keep choice), (vii) onset of all the choices of fair trials (5:5, for punishment, help and keep choice), (viii) onset of all the transfers of fair trials (5:5, for punishment, help and keep choice). The GLM additionally included six movement regressors of no interest, three for translational movements (x, y, z) and three for rotation movements (pitch, roll, yaw). All regressors were convolved with the canonical hemodynamic response function. Individual contrast images (for “Punishment”, “Help”, “Punishment-Help”) were transferred to a second-level random-effects analysis using two-sample t-tests to compare the stress and control groups. More specifically, several participants were excluded in the second level because coefficients for the parameters could not be estimated when participants never, or only one time, choosing help or punishment per functional run. To this end, the group contrast of “Punishment-Help” was computed with 43 participants (stress group: 22; and control group:21), the group contrast of “Punishment” was computed with 50 participants (stress group: 26; and control group:24), the group contrast of “Help” was computed with 45 participants (stress group: 23; and control group:22).

**In the third model (GLM_ Utility)**, we computed a GLM with a parametric design to identify brain regions coding “Utility” on the transfer window when participants spending chips (MU) to punish the proposer or help the recipient. The Utility was defined in our “Other-regarding inequality aversion model”. Four regressors were included in the GLM in the following order: (i) onset of the first transfer stage, (ii) parametric modulation of the trial-wise “Utility”, (iii) onset of the decision stage at the beginning of each trial, and (iv) onset of the second transfer stage. Same as the first model, we modeled BOLD responses at these onsets as stick functions. All regressors and six head movement regressors of no interest were convolved with a canonical hemodynamic response function. For each event, onset regressor parameter estimates were obtained and contrast images of each of the parameters against zero were generated. The obtained images were transferred to a second-level random-effects analysis using two-sample t tests and conjunction analysis to compare the stress and control groups.

**Functional connectivity analysis (gPPI analysis)**

To investigate whether the functional connectivity of the amygdala differed during help and punishing decisions and whether it was affected by stress, we performed a whole-brain gPPI analysis with the right amygdala as seed region5. The location of the right amygdala seed ROI was based on a 6 mm radius sphere centered at the peak activation within the contrasts of stress vs. control (GLM_ Inequity). We estimated a GLM with the following regressors: (i) a physiological regressor (i.e., the entire time series of the seed region over the whole experiment), (ii) a psychological regressor for the onset of the punishment choices, (iii) the PPI regressor for the punishment choices, (iv) a psycho-logical regressor for the onset of the help choices, and (v) a PPI regressor for the help choices. The onset and PPI regressors were convolved with a canonical form of the hemodynamic response. The model also included the six motion parameters as regressors of no interest. Individual contrast images for functional connectivity (“punishment”, “help”, “punishment vs. help”) were transferred to a second-level random-effects analysis using a two-sample t test and one-sample t test. We performed whole-brain corrections for multiple comparisons at the cluster level (P _FWE_ < 0.05), the individual voxel threshold was set to P < 0.001. All reported coordinates (x, y, z) are in MNI space.

**References**

[1] Riccio, D. C., Ackil, J., & Burch-Vernon, A. (1992). Forgetting of stimulus attributes: Methodological implications for assessing associative phenomena. *Psychological Bulletin*. <https://doi.org/10.1037/0033-2909.112.3.433>

[2] Hu, Y., Strang, S., & Weber, B. (2015). Helping or punishing strangers: Neural correlates of altruistic decisions as third-party and of its relation to empathic concern. *Frontiers in Behavioral Neuroscience*, *9*(FEB), 24. https://doi.org/10.3389/fnbeh.2015.00024

[3] Wood, A. P., Dawe, S., & Gullo, M. J. (2013). The role of personality, family influences, and prosocial risk-taking behavior on substance use in early adolescence. *Journal of Adolescence*, *36*(5), 871–881. <https://doi.org/10.1016/j.adolescence.2013.07.003>

[4] Cohen, S., Kamarck, T., & Mermelstein, R. (1983). Perceived Stress Scale. In *Journal of Health and Social Behavior*.

[5] Grös, D. F., Antony, M. M., Simms, L. J., & McCabe, R. E. (2007). Psychometric Properties of the State-Trait Inventory for Cognitive and Somatic Anxiety (STICSA): Comparison to the State-Trait Anxiety Inventory (STAI). *Psychological Assessment*. https://doi.org/10.1037/1040-3590.19.4.369

[6] Murphy, R. O., Ackermann, K. A., & Handgraaf, M. J. J. (2011). Measuring Social Value Orientation. *Judgment and Decision Making*. https://doi.org/10.2139/ssrn.1804189

[7] Vandeweghe, L., Matton, A., Beyers, W., Vervaet, M., Braet, C., & Goossens, L. (2016). Psychometric properties of the BIS/BAS scales and the SPSRQ in flemish adolescents. *Psychologica Belgica*, *56*(4), 406–420. <https://doi.org/10.5334/pb.298>

[8] Davis, M. H. (1983). Measuring individual differences in empathy: Evidence for a multidimensional approach. *Journal of Personality and Social Psychology*. https://doi.org/10.1037/0022-3514.44.1.113

[9] Philippe Rushton, J., Chrisjohn, R. D., & Cynthia Fekken, G. (1981). The altruistic personality and the self-report altruism scale. *Personality and Individual Differences*. https://doi.org/10.1016/0191-8869(81)90084-2
