# Supplementary material for: Acute stress during witnessing injustice shifts third-party interventions from punishing the perpetrator to helping the victim
Source: PLoS Biol. 2024 May 16;22(5):e3002195. doi: 10.1371/journal.pbio.3002195 (PMC11098560; doi:10.1371/journal.pbio.3002195)
Supplement: S1 Fig — (DOCX) [file pbio.3002195.s002.docx]

Fig. S1.

**Acute stress modulated the prosocial preference.**

We compared the punishment, help and selfish choices, as a function of the fair condition (50:50, 60:40, 70:30, 80:20, 90:10). As expected, we found that as the degree of unfairness increased, the proportion of punishment and help behavior increased, and the proportion of selfish behavior decreased correspondingly and this effect was similar in the stress and control group (**Fig S1A&B**, stress main effect of choice rate: F (1, 50) = 0.71, P = 0.40, *η*_p_^2^ = 0.014; stress main effect of contribution: F (1, 50) = 1.04, P = 0.31, *η*_p_^2^ = 0.020). The source data of Fig S1A, Fig S1B can be found at https://osf.io/fkae9/.
