# Supplementary material for: Acute stress during witnessing injustice shifts third-party interventions from punishing the perpetrator to helping the victim
Source: PLoS Biol. 2024 May 16;22(5):e3002195. doi: 10.1371/journal.pbio.3002195 (PMC11098560; doi:10.1371/journal.pbio.3002195)
Supplement: S2 Fig — (DOCX) [file pbio.3002195.s003.docx]

Fig. S2.

**Model validation and Parameter recovery.**

The posterior predictive check further showed a high correlation between the actual behaviors and model prediction (**Fig S2 A&B**, r = 0.82, p < 0.001 in punishment contribution and r = 0.75, p < 0.001 in help contribution), which suggests that our model can predict participants’ behavior well. The recovery analysis revealed that the correspondence between the true and recovered parameters was significantly high for two free parameters (**Fig S2 C&D,** *α*: *r* = 0.986, *p* < 0.001; *β*: *r* = 0.976, *p* < 0.001)**.**

Furthermore, the model predicted punishment bias (difference of the two free parameters: α-β) was positively related to the actual punishment rate and contributions, (**Fig S2E& S2F**, r = 0.76, *P* < 0.01 for punishment rate and r = 0.66, *P* < 0.01 for punishment contributions), but negatively related to the actual help rate and contributions (r = -0.67, *P* < 0.01 for help rate and r = -0.48, *P* < 0.01 for help contributions). The source data of Fig S2A-Fig S2F can be found at https://osf.io/fkae9/.
