# Supplementary material for: Acute stress during witnessing injustice shifts third-party interventions from punishing the perpetrator to helping the victim
Source: PLoS Biol. 2024 May 16;22(5):e3002195. doi: 10.1371/journal.pbio.3002195 (PMC11098560; doi:10.1371/journal.pbio.3002195)
Supplement: S3 Fig — (DOCX) [file pbio.3002195.s004.docx]

Fig. S3.

******

**Stress influences the neural correlates of punishment versus help decision under all unfair conditions (relative and unfair condition, 60:40~90:10).**

We also regressed the fMRI time series into a general linear model (GLM_ Choice2) to investigate how acute stress affected the brain’s decision circuitry. In the GLM (GLM_ Choice2), we aimed to recognize brain regions whose behavior was associated with punishment and help choice under all the unfair conditions (60:40~90:10). We defined the following two onset regressors of interest: (i) onset of the punishment choice of all unfair trials, (ii) onset of the help choice of all unfair trials. We also defined the following uninterested regressors: (iii) onset of the punishment transfer of all unfair trials, (iv) onset of the help transfer of all unfair trials, (v) onset of all the choices of fair trials (5:5, for punishment, help and keep choice), (vi) onset of all the transfers of fair trials (5:5, for punishment, help and keep choice). The GLM additionally included six movement regressors of no interest, three for translational movements (x, y, z) and three for rotation movements (pitch, roll, yaw). All regressors were convolved with the canonical hemodynamic response function. Individual contrast images (for “Punishment”, “Help”) were transferred to a second-level analysis using 2 (group) by 2 (Choice) mixed factorial analysis of variance (ANOVA) ^[1]^. More specifically, several participants were excluded in the second level because coefficients for the parameters could not be estimated when participants never, or only one time, choosing help or punishment per functional run. To this end, the group contrast was computed with 48 participants (stress group: 25; and control group:23), By comparing trials in which punishment is selected vs trials in which help is selected between stress and control group under all unfair conditions, we found that stress induced a higher activity in right DLPFC, PCC and right Thalamus when select punishment choices relative to help choices (Figure S3, initial threshold set at *P* _uncorrected_ < 0.001, whole-brain cluster corrected at P _FWE_ < 0.05). The source data of Fig S3 can be found at https://osf.io/fkae9/.

**Reference**

[1] Qin, S., Hermans, E. J., van Marle, H. J. F., Luo, J., & Fernández, G. (2009). Acute Psychological Stress Reduces Working Memory-Related Activity in the Dorsolateral Prefrontal Cortex. *Biological Psychiatry*, *66*(1), 25–32. <https://doi.org/10.1016/j.biopsych.2009.03.006>
