# Supplementary material for: Acute stress during witnessing injustice shifts third-party interventions from punishing the perpetrator to helping the victim
Source: PLoS Biol. 2024 May 16;22(5):e3002195. doi: 10.1371/journal.pbio.3002195 (PMC11098560; doi:10.1371/journal.pbio.3002195)
Supplement: S1 Table — (DOCX) [file pbio.3002195.s005.docx]

Table S1.

Control variables per group [M (SD)].

|  | **Stress** | **Control** |
| --- | --- | --- |
| **PSS** | 25.38(1.43) | 24.12(0.99) |
| **STICSA** | 16.40(0.97) | 15.66(0.96) |
| **SVO** | 34.64(1.63) | 34.92(1.66) |
| **BIS** | 2.65(0.10) | 2.73(0.07) |
| **BAS** | 2.94(0.10) | 3.13(0.08) |
| **IRI** | 2.52(0.07) | 2.58(0.06) |
| **APS** | 58.35(3.54) | 63.64(2.92) |
| **Base Testosterone** | 2.06(0.14) | 2.16(0.12) |
| **Base Oxytocin** | 17.20(0.83) | 17.69(0.84) |

**Psychological and personality measures:** Before the experiment, participants completed an online survey (implemented via Qualtrics software, 2009, Provo, UT, USA. https://www.qualtrics.com), which included demographic questions and some personality measures: Perceived Stress Scale (PSS) ^[1]^; State-Trait Inventory for Cognitive and Somatic Anxiety (STICSA) ^[2]^; Social Value Orientation (SVO) ^[3]^; Behavioral Inhibition System and Behavioral Activation System Scales (BIS/BAS Scales) ^[4]^; Interpersonal Reactivity Index (IRI) ^[5]^; Altruistic personality scale (APS) ^[6]^. We found no group differences in any of these variables (all *P* > 0.131).

**References**

[1] Cohen, S., Kamarck, T., & Mermelstein, R. (1983). Perceived Stress Scale. In *Journal of Health and Social Behavior*.

[2] Grös, D. F., Antony, M. M., Simms, L. J., & McCabe, R. E. (2007). Psychometric Properties of the State-Trait Inventory for Cognitive and Somatic Anxiety (STICSA): Comparison to the State-Trait Anxiety Inventory (STAI). *Psychological Assessment*. https://doi.org/10.1037/1040-3590.19.4.369

[3] Murphy, R. O., Ackermann, K. A., & Handgraaf, M. J. J. (2011). Measuring Social Value Orientation. *Judgment and Decision Making*. https://doi.org/10.2139/ssrn.1804189

[4] Vandeweghe, L., Matton, A., Beyers, W., Vervaet, M., Braet, C., & Goossens, L. (2016). Psychometric properties of the BIS/BAS scales and the SPSRQ in flemish adolescents. *Psychologica Belgica*, *56*(4), 406–420. <https://doi.org/10.5334/pb.298>

[5] Davis, M. H. (1983). Measuring individual differences in empathy: Evidence for a multidimensional approach. *Journal of Personality and Social Psychology*. https://doi.org/10.1037/0022-3514.44.1.113

[6] Philippe Rushton, J., Chrisjohn, R. D., & Cynthia Fekken, G. (1981). The altruistic personality and the self-report altruism scale. *Personality and Individual Differences*. https://doi.org/10.1016/0191-8869(81)90084-2
