# Supplementary material for: Acute stress during witnessing injustice shifts third-party interventions from punishing the perpetrator to helping the victim
Source: PLoS Biol. 2024 May 16;22(5):e3002195. doi: 10.1371/journal.pbio.3002195 (PMC11098560; doi:10.1371/journal.pbio.3002195)
Supplement: S2 Table — (DOCX) [file pbio.3002195.s006.docx]

Table S2.

**Stress-induced differences in the brain among all fair conditions in the decision stage.**

|  |  | **MNI Coordinates** | | | **Z score** | **voxels** |
| --- | --- | --- | --- | --- | --- | --- |
| **Brain region and contrast** | **Side** | **X** | **Y** | **Z** |  |  |
| **Stress > Control** | | | | | | |
| Cerebellum | R | 24 | -74 | -20 | 4.44 | 288 |
| Superior Temporal Gyrus | R | 66 | -22 | 14 | 4.00 | 165 |
| Insula (SVC)* | L | -36 | -4 | -8 | 3.54 | 51 |
| Insula (SVC)* | R | 46 | 10 | 2 | 3.29 | 8 |
| Amygdala (SVC)* | L | -28 | -4 | -22 | 3.19 | 1 |
| **Control > Stress** |  | | | | | |
| **-** |  |  |  |  |  |  |

Initial whole-brain threshold at *P* <0.001 uncorrected and cluster corrected at *P* < 0.05 FWE.

The regions of insula were the peaks for a small volume correction (SVC) within an 8-mm spherical ROI on MNI x, y, z = 38, 18, 0 and MNI x, y, z = -38, 14, 4 as in previous studies ^[1]^. The regions of Amygdala were small volume corrected at p<0.001 uncorrected and cluster corrected at *P* < 0.05 FWE, the anatomical masker of left amygdala was created using the SPM Wake Forest University (WFU) Pickatlas toolbox (www.ansir.wfubmc.edu, version 3.0).

**Reference**

[1] Kogler, L., Müller, V. I., Chang, A., Eickhoff, S. B., Fox, P. T., Gur, R. C., & Derntl, B. (2015). Psychosocial versus physiological stress - Meta-analyses on deactivations and activations of the neural correlates of stress reactions. *NeuroImage*, *119*, 235–251. <https://doi.org/10.1016/j.neuroimage.2015.06.059>
