# Supplementary material for: Acute stress during witnessing injustice shifts third-party interventions from punishing the perpetrator to helping the victim
Source: PLoS Biol. 2024 May 16;22(5):e3002195. doi: 10.1371/journal.pbio.3002195 (PMC11098560; doi:10.1371/journal.pbio.3002195)
Supplement: S3 Table — (DOCX) [file pbio.3002195.s007.docx]

Table S3.

**Neural response for increasing distributional inequity |Proposer-Recipient | in decision stage.**

|  |  | MNI Coordinates | | | Z score | voxels |
| --- | --- | --- | --- | --- | --- | --- |
| Brain region and contrast | Side | X | Y | Z |  |  |
| Control > Stress | | | | | | |
| Amygdala (SVC)* | R | 26 | -2 | -14 | 3.38 | 6 |
| Stress > Control | | | | | | |
| - |  |  |  |  |  |  |
| Conjunction |  | | | | | |
| Precentral Gyrus | R | 38 | -22 | 54 | 4.01 | 185 |
| TPJ | R | 50 | -44 | 58 | 5.36 | 1251 |
| Middle Temporal Gyrus | R | 58 | -34 | -16 | 4.71 | 361 |
| Inferior Frontal Gyrus | R | 52 | 18 | 6 | 4.49 | 281 |
| Cerebellum | L | -44 | -68 | -36 | 4.39 | 195 |
| DLPFC  (Superior Frontal Gyrus) | R | 22 | 56 | 36 | 4.30 | 86 |
| VLPFC  (Middle Frontal Gyrus) | R | 24 | 40 | -16 | 4.10 | 537 |
| DLPFC  (Middle Frontal Gyrus) | R | 36 | 26 | 48 | 4.00 | 162 |

Inequity was defined as “δ = |MU Proposer – MU Recipient|.

Initial whole-brain threshold at *P* < 0.001 uncorrected and cluster corrected at *P* < 0.05 FWE.

Small volume correction (SVC)* based on anatomically defined bilateral amygdala region of interests (ROIs), and FWE corrected *P* < 0.05. TPJ, temporo-parietal junction. DLPFC, dorsolateral prefrontal cortex. VLPFC, ventrolateral prefrontal cortex.

Note: We further examined additional regions implicated in inequity processing in previous studies ^[1]^. These regions including follows (MNI coordinates), however, none of these brain regions yielded significant results in the “Stress-Control”/ “Control-Stress”/ “Stress Conjunction Control” conditions after correcting for multiple comparisons (SVC corrected *P*_FWEs_ > 0.05).

Left dorsal ACC [-4, 16, 48]

Right dorsal ACC [8, 22, 40]

Right AI [38, 20, 0]

Left AI [-30, 24, 2]

Right putamen [22, 12, 2]

Left putamen [-22, 12, 2]

Right vlPFC [34, 21, -18]

Left vlPFC [-44, 24, -8]

Right dlPFC (middle frontal gyrus/sfg) [40, 36, 26]

Left dlPFC (middle frontal gyrus) [-30, 38, 30]

Right dmPFC [8, 60, 16]

**Reference**

[1] Feng, C., Luo, Y. J., & Krueger, F. (2015). Neural signatures of fairness-related normative decision making in the ultimatum game: a coordinate-based meta-analysis. *Human brain mapping*, *36*(2), 591–602. <https://doi.org/10.1002/hbm.22649>
