# Supplementary material for: Acute stress during witnessing injustice shifts third-party interventions from punishing the perpetrator to helping the victim
Source: PLoS Biol. 2024 May 16;22(5):e3002195. doi: 10.1371/journal.pbio.3002195 (PMC11098560; doi:10.1371/journal.pbio.3002195)
Supplement: S4 Table — (DOCX) [file pbio.3002195.s008.docx]

Table S4.

Neural Correlates of utility in Transfer stage

|  |  | **MNI Coordinates** | | | **Z score** | **voxels** |
| --- | --- | --- | --- | --- | --- | --- |
| **Brain region and contrast** | **Side** | **X** | **Y** | **Z** |  |  |
| **Control > Stress** | | | | | | |
| **Stress > Control -** | | | | | | |
| **Conjunction** |  | | | | | |
| Precentral Gyrus | R | 36 | -24 | 54 | 5.25 | 831 |
| Fusiform Gyrus | L | -8 | -66 | -4 | 5.13 | 340 |
| **VMPFC**  (Medial Frontal Gyrus) | L | -6 | 32 | -8 | 4.58 | 1039 |
| Middle Temporal Gyrus | R | 60 | -54 | 12 | 4.40 | 157 |
| Insula | R | 46 | -20 | 20 | 4.38 | 185 |
| Middle Temporal Gyrus | L | -56 | -16 | -8 | 4.36 | 408 |
| **Posterior Cingulate** | L | -6 | -54 | 18 | 4.34 | 297 |
| Cuneus | R | 12 | -88 | 28 | 4.31 | 654 |
| Superior Temporal Gyrus | L | -60 | -60 | 24 | 4.24 | 312 |
| Middle Temporal Gyrus | R | 64 | -18 | -6 | 3.69 | 180 |
| DLPFC  (Middle Frontal Gyrus) | R | 34 | 32 | 28 | 4.30 | 358 |

Initial whole-brain threshold at P <0.001 uncorrected and cluster corrected at P < 0.05 FWE.

VMPFC, ventromedial prefrontal cortex. DLPFC, dorsolateral prefrontal cortex.

Note: We further examined additional regions implicated in stress processing including amygdala, insula and ACC in the contrast of Stress > Control and Control > Stress through small-volume correction (SVC), and we found that none of these brain regions yielded significant results (SVC corrected *P*_FWEs_ > 0.05). We defined the anatomical ROIs for the bilateral amygdala, anterior cingulate cortex and insula using the SPM Wake Forest University (WFU) Pickatlas toolbox (www.ansir.wfubmc.edu, version 3.0) ^[1]^.

Reference

[1] Tzourio-Mazoyer, N., Landeau, B., Papathanassiou, D., Crivello, F., Etard, O., Delcroix, N., Mazoyer, B., & Joliot, M. (2002). Automated anatomical labeling of activations in SPM using a macroscopic anatomical parcellation of the MNI MRI single-subject brain. *NeuroImage*, *15*(1), 273–289. https://doi.org/10.1006/nimg.2001.0978
