# Supplementary material for: Acute stress during witnessing injustice shifts third-party interventions from punishing the perpetrator to helping the victim
Source: PLoS Biol. 2024 May 16;22(5):e3002195. doi: 10.1371/journal.pbio.3002195 (PMC11098560; doi:10.1371/journal.pbio.3002195)
Supplement: S5 Table — (DOCX) [file pbio.3002195.s009.docx]

Table S5.

**PPI: Regions showing stress group functional connectivity with the right** **amygdala when making a punishment choice in the unfair condition.**

|  |  | **MNI Coordinates** | | | **Z score** | **voxels** |
| --- | --- | --- | --- | --- | --- | --- |
| **Brain region and contrast** | **Side** | **X** | **Y** | **Z** |  |  |
| **VMPFC**  **Superior Medial Gyrus** | L | -6 | 46 | 2 | 4.42 | 336 |
| Middle Temporal Gyrus | R | 54 | -64 | 10 | 4.74 | 127 |
| Middle Temporal Gyrus | L | -64 | -50 | 8 | 4.50 | 502 |
| Middle Frontal Gyrus | R | 38 | -2 | 64 | 4.49 | 214 |
| Superior Medial Gyrus | L | -4 | 50 | 38 | 4.39 | 139 |
| IFG (p. Triangularis) | R | 54 | 16 | 12 | 4.38 | 133 |
| Superior Temporal Gyrus | L | -60 | -2 | 4 | 4.18 | 124 |
| Middle Occipital Gyrus | R | 36 | -90 | 14 | 4.13 | 270 |
| Rolandic Operculum | R | 60 | -4 | 18 | 4.12 | 102 |
| MCC | L | -2 | 6 | 64 | 3.95 | 138 |
| Linual Gyrus | R | 6 | -64 | 10 | 3.86 | 95 |
| Precentral Gyrus | L | -48 | 4 | 44 | 3.81 | 127 |
| Cuneus | L | -8 | -84 | 22 | 3.66 | 96 |

Initial whole-brain threshold at P <0.001 uncorrected and cluster corrected at P< 0.05 FWE. VMPFC, ventromedial prefrontal cortex. IFG, inferior frontal gyrus. MCC, medial cingulate cortex.
