# Supplementary material for: Acute stress during witnessing injustice shifts third-party interventions from punishing the perpetrator to helping the victim
Source: PLoS Biol. 2024 May 16;22(5):e3002195. doi: 10.1371/journal.pbio.3002195 (PMC11098560; doi:10.1371/journal.pbio.3002195)
Supplement: S6 Table — (DOCX) [file pbio.3002195.s010.docx]

Table S6.

**Stress induced higher neural correlates of relative severity in punishment versus help (α-β).**

|  |  | **MNI Coordinates** | | | **Z score** | **voxels** |
| --- | --- | --- | --- | --- | --- | --- |
| **Brain region and contrast** | **Side** | **X** | **Y** | **Z** |  |  |
| **Control > Stress -** | | | | | | |
| **Stress > Control** | | | | | | |
| Posterior Cingulate | R | 2 | -40 | 24 | 5.26 | 141 |
| Angular | R | 44 | -60 | 40 | 3.99 | 121 |
| Anterior Cingulate (SVC)* | R | 2 | 36 | 16 | 4.34 | 85 |

Initial whole-brain threshold at P <0.001 uncorrected and cluster corrected at P < 0.05 FWE.

Small volume correction (SVC)* based on anatomically defined bilateral Anterior Cingulate region of interests (ROIs), and FWE corrected P < .05.

Note: We further examined additional regions implicated in stress processing including amygdala and insula in the contrast of Stress > Control and Control > Stress through small-volume correction (SVC), and we found that none of these brain regions yielded significant results (SVC corrected *P*_FWEs_ > 0.05). We defined the anatomical ROIs for the bilateral amygdala and insula using the SPM Wake Forest University (WFU) Pickatlas toolbox.
