# Supplementary material for: Acute stress during witnessing injustice shifts third-party interventions from punishing the perpetrator to helping the victim
Source: PLoS Biol. 2024 May 16;22(5):e3002195. doi: 10.1371/journal.pbio.3002195 (PMC11098560; doi:10.1371/journal.pbio.3002195)
Supplement: S7 Table — (DOCX) [file pbio.3002195.s011.docx]

Table S7.

**Linear Relationship between Stress-Related Variance and Behavioral Decision Making (Help Rate)**

| Dependent: rate of help |  | unit | value | Coefficient (univariable) | Coefficient (multivariable) | Coefficient (final) |
| --- | --- | --- | --- | --- | --- | --- |
| Z_Cortisol (AUCI) | [-2.1,3.1] | Mean ± SD | 0.0 ± 1.0 | 0.09 (0.01 to 0.16, p=.027) | 0.09 (0.01 to 0.18, p=.038) | 0.09 (0.01 to 0.16, p=.027) |
| Z_Heart Rate (delta) | [-1.6,2.6] | Mean ± SD | 0.0 ± 1.0 | 0.04 (-0.03 to 0.12, p=.271) | 0.02 (-0.07 to 0.11, p=.664) |  |
| Z_Negative_T2-T1 | [-3.6,2.7] | Mean ± SD | 0.0 ± 1.0 | -0.01 (-0.08 to 0.07, p=.854) | -0.05 (-0.13 to 0.04, p=.264) |  |
|  | | | | | | |
