# Supplementary material for: Acute stress during witnessing injustice shifts third-party interventions from punishing the perpetrator to helping the victim
Source: PLoS Biol. 2024 May 16;22(5):e3002195. doi: 10.1371/journal.pbio.3002195 (PMC11098560; doi:10.1371/journal.pbio.3002195)
Supplement: S8 Table — (DOCX) [file pbio.3002195.s012.docx]

Table S8.

**Linear Relationship between Stress-Related Variance and Behavioral Decision Making (Pinish Rate)**

| Dependent: rate of punishment |  | unit | value | Coefficient (univariable) | Coefficient (multivariable) | Coefficient (final) |
| --- | --- | --- | --- | --- | --- | --- |
| Z_AUCI | [-2.1,3.1] | Mean ± SD | 0.0 ± 1.0 | -0.09 (-0.17 to -0.02, p=.017) | -0.10 (-0.19 to -0.01, p=.026) | -0.09 (-0.17 to -0.02, p=.017) |
| Z_HR_delta | [-1.6,2.6] | Mean ± SD | 0.0 ± 1.0 | -0.04 (-0.12 to 0.03, p=.257) | -0.02 (-0.11 to 0.07, p=.709) |  |
| ZNegative_T2_T1 | [-3.6,2.7] | Mean ± SD | 0.0 ± 1.0 | 0.00 (-0.08 to 0.08, p=.934) | 0.04 (-0.04 to 0.13, p=.293) |  |
|  | | | | | | |
