# Supplementary material for: Acute stress during witnessing injustice shifts third-party interventions from punishing the perpetrator to helping the victim
Source: PLoS Biol. 2024 May 16;22(5):e3002195. doi: 10.1371/journal.pbio.3002195 (PMC11098560; doi:10.1371/journal.pbio.3002195)
Supplement: S9 Table — (DOCX) [file pbio.3002195.s013.docx]

Table S9. The neural correlates of total utility associated with aversion to witnessing someone else harm others (α).

|  |  | **MNI Coordinates** | | | **Z score** | **voxels** |
| --- | --- | --- | --- | --- | --- | --- |
| **Brain region and contrast** | **Side** | **X** | **Y** | **Z** |  |  |
| **Control > Stress** | | | | | | |
| No region passed the threshold.  **Stress > Control** | | | | | | |
| Precuneus* | R | 16 | -56 | 46 | 5.30 | 460 |
| Angular* | R | 46 | -60 | 40 | 4.44 | 124 |
| Middle Frontal Gyrus* | R | 44 | 50 | 4 | 4.43 | 114 |
| Cerebellum^#^ | L | -38 | -74 | -44 | 4.59 | 41 |
| **Conjunction** |  |  |  |  |  |  |
| No common region found |  |  |  |  |  |  |

*Initial whole-brain threshold at P <0.001 uncorrected at voxel level and cluster corrected at P < 0.05 FWE.

^#^Initial whole-brain threshold at P _FDR_ <0.001 at voxel level and cluster corrected at P < 0.05 FWE.
