# Supplementary material for: Acute stress during witnessing injustice shifts third-party interventions from punishing the perpetrator to helping the victim
Source: PLoS Biol. 2024 May 16;22(5):e3002195. doi: 10.1371/journal.pbio.3002195 (PMC11098560; doi:10.1371/journal.pbio.3002195)
Supplement: S10 Table — (DOCX) [file pbio.3002195.s014.docx]

Table S10. The neural correlates of total utility associated with aversion to witnessing someone else being harmed (β).

|  |  | **MNI Coordinates** | | | **Z score** | **voxels** |
| --- | --- | --- | --- | --- | --- | --- |
| **Brain region and contrast** | **Side** | **X** | **Y** | **Z** |  |  |
| **Stress > Control** | | | | | | |
| Precuneus | L | -26 | -56 | 52 | 3.83 | 31 |
| **Control > Stress** |  |  |  |  |  |  |
| Anterior Cingulate | L | 0 | 36 | 16 | 3.67 | 57 |
| **Conjunction**  No common region found |  |  |  |  |  |  |

Note: The regions listed above did not pass the FWE-corrected threshold.

The threshold we set was: Initial whole-brain threshold at P <0.001 uncorrected and cluster corrected at P < 0.05 FWE.
