# Supplementary material for: Acute stress during witnessing injustice shifts third-party interventions from punishing the perpetrator to helping the victim
Source: PLoS Biol. 2024 May 16;22(5):e3002195. doi: 10.1371/journal.pbio.3002195 (PMC11098560; doi:10.1371/journal.pbio.3002195)
Supplement: Attachment — Submitted filename: Response to Reviewers.docx [file pbio.3002195.s015.docx]

**Editorial Comments:**

As you will see below, the reviewers agree that the study is overall interesting and potentially provides important insights. However, R1 raises concerns about empathy as a potential confounder and identifies other concerns about the computational modelling and the neuroimaging analysis. R2 has less serious concerns but mentions some previous studies that might impact novelty of this study. R3 raise concerns about the integration of the previous literature and some technical aspects about the computational and neuroimaging aspects of the study. Please bear the reviewer comments carefully in mind when revising the manuscript.

Please also note that we had asked the reviewers if they want to provide additional feedback after reading the other reviewers' reports. In this context, Reviewer 3 wrote:

"We don’t agree with Reviewer 1’s comments on the computational modeling: 1) inequity is included in the full model (equation 1) because the payoffs of both players are included in the utility such that a higher inequity between the two players prompts the third-party to punish the transgressor more harshly and help the victim more, and 2) the authors used RSS to fit the model parameters but AIC to compare the models which takes care of the number of parameters."

We usually use these comments only to inform the discussion between the editorial team and the academic editors but in this case we thought this comment would be useful for you when revising your manuscript and asked the reviewer for permission to share these comments with you.

**Response:** We are very grateful for the thoughtful and constructive comments raised by the editor and three reviewers. We have carefully addressed all of these comments and have also revised the manuscript accordingly. The followings are our rebuttal responses to their comments and concerns. The responses and important changes in the revised manuscript were colored in blue.

**Response to Reviewer #1**

General comment：

Wang et al. carried out an fMRI experiment with a newly designed third-party intervention game. They aimed to study the impact of the cold pressure test (CPT) on the intervention behavior and fMRI signals of the third party, utilizing a computational model. The authors discovered that CPT lowered the third party's readiness to penalize norm violators (proposers in unfair divisions) and the intensity of the penalty. However, it elevated their willingness to aid recipients. These behavioral patterns were further interpreted by their computational model.

In their fMRI analysis, Wang et al. first reported that the activity in the left amygdala (though the right amygdala exhibited an opposite pattern), bilateral insula, and rTPJ, correlated with the inequity between the proposer and recipient, was larger in the CPT group than in the control group. Nevertheless, rTPJ, VLPFC, and DLPFC were similar in both groups. Their subsequent PPI and mediation analyses revealed that in the CPT group, the functional connectivity of the right amygdala with the vmPFC was higher when choosing the punishment option, and CPT reduced the punishment rate by enhancing amygdala-vmPFC connectivity. Contrarily, DLPFC activity displayed a reverse pattern in the CPT and control groups, with higher DLPFC activity when choosing the punishment and help options, respectively. They also found that the correlation between punishment severity bias and the activity of ACC, PC, and rTPJ was stronger in CPT versus control participants.

The authors interpreted these results as showing that "acute stress decreases third-party's willingness to punish violators and the severity of the punishment, but increases their willingness to help victims by using an intuitive mode suggested by higher activity in the insula, amygdala, and TPJ, and smaller involvement of the amygdala-vmPFC network, DLPFC activity, and theory-of-mind network." If this interpretation is robustly validated, this study could provide a significant insight into social neuroscience.

However, the reviewer believes there are serious concerns in the areas of behaviors, computational modeling, and fMRI analysis. These need to be addressed to make the authors' interpretations persuasive. These are listed as follows:

**Response：**

Thank you for your thorough and well-structured summary of our study. We appreciate your acknowledgement of our work and your meticulous review of our suggestions. Your constructive feedback has greatly enhanced the rigor of our findings across various aspects, including behavioral analysis, fMRI, computational modeling, and writing. We have now carefully addressed each of your comments and suggestions, ensuring that we incorporate the necessary improvements. It is possible that some of the revised sections may still have room for further enhancement, and we will be attentive to such opportunities. Once again, we are grateful for your time and effort in correcting our research.

**Comment 1.1:** Behaviors.

The authors appear to presume that behavioral changes were a result of acute stress, particularly the impact of cortisol and the hypothalamic-pituitary-adrenal (HPA) axis. However, subjective and cognitive experiences of a negative event (even reading a story) can increase empathy. Some research has suggested that individuals who have faced adversities are more likely to exhibit helping behaviors (e.g., Decety, J. Ann N Y Acad Sci. 2011).
To counter this possibility, the authors need to separate the effects of cortisol increase, heart rate increase, and the stress questionnaire and show that the cortisol increase has the strongest influence on behavioral changes. Such analysis is possible because the correlation coefficient among these three stress variables is generally not very high (some show a clear increase in cortisol concentration, while others do not).

**Response：**

Thank you very much for your suggestion. We hypothesize that behavioral changes result from acute stress. Acute stress is accompanied by rapid sympathetic changes, such as heart rate fluctuations, as well as slower changes in the HPA axis. However, we believe that changes in the HPA axis play a more significant role due to their prolonged effects on the brain and hormones. Moreover, since heart rate, cortisol levels, and mood changes are all outcomes of acute stress and exhibit correlation (refer to the correlation matrix: Figure R 1.1.1), we conducted both one-way regression and multifactorial regression analyses. The simple regression analysis aimed to establish the relationship between a single indicator and behavioral changes. On the other hand, the multifactorial analysis enabled us to indirectly determine, based on the regression coefficients, which factor plays a more influential role. Our findings from both the univariate and multivariate regression analyses revealed that cortisol emerged as a significant predictor of the rate of help and punishment (refer to Table R 1.1.1 and R 1.1.2). In contrast, heart rate and negative affect did not demonstrate significant predictive power in relation to behavioral decisions. These results suggest that cortisol, as a stress indicator, holds greater importance in influencing participants’ behavioral choices. Regarding this part of the results, we have added in the revised version.

*In addition, we conducted multivariate regression analyses to investigate the primary factor influencing stress-induced behavioral variation. Our findings revealed that cortisol played a significant role in shaping individual’s behaviors. Specifically, we observed a positive correlation between cortisol levels and the proportion of helpfulness (β = 0.09，p = 0.027). Conversely, there was a negative correlation between cortisol levels and the proportion of punishment (β = -0.09，p = 0.017) (****Table S7& Table S8****).(page 5, line 187)*

Figure R 1.1.1 The correlation of Cortisol, Heart rate, and Negative emotion following acute stress. Cortisol levels were determined by calculating the area under the curve based on cortisol values over time. Heart rate was calculated as the difference between the heart rate during the Cold Pressor Test (CPT) period and the baseline heart rate. Negative mood was derived from the average score of negative-related items in the PANAS scale, also calculated by comparing the CPT period to the baseline heart rate. Additionally, all three-stress metrics were standardized to Z scores for comparison purposes.

**Table R1.1.1: Linear Relationship between Stress-Related Variance and Behavioral Decision Making (Help Rate)**

| Dependent: rate of help |  | unit | value | Coefficient (univariable) | Coefficient (multivariable) | Coefficient (final) |
| --- | --- | --- | --- | --- | --- | --- |
| Z_Cortisol (AUCI) | [-2.1,3.1] | Mean ± SD | 0.0 ± 1.0 | 0.09 (0.01 to 0.16, p=.027) | 0.09 (0.01 to 0.18, p=.038) | 0.09 (0.01 to 0.16, p=.027) |
| Z_Heart Rate (delta) | [-1.6,2.6] | Mean ± SD | 0.0 ± 1.0 | 0.04 (-0.03 to 0.12, p=.271) | 0.02 (-0.07 to 0.11, p=.664) |  |
| Z_Negative_T2-T1 | [-3.6,2.7] | Mean ± SD | 0.0 ± 1.0 | -0.01 (-0.08 to 0.07, p=.854) | -0.05 (-0.13 to 0.04, p=.264) |  |
|  | | | | | | |

**Table R1.1.2: Linear Relationship between Stress-Related Variance and Behavioral Decision Making (Pinish Rate)**

| Dependent: rate of punishment |  | unit | value | Coefficient (univariable) | Coefficient (multivariable) | Coefficient (final) |
| --- | --- | --- | --- | --- | --- | --- |
| Z_AUCI | [-2.1,3.1] | Mean ± SD | 0.0 ± 1.0 | -0.09 (-0.17 to -0.02, p=.017) | -0.10 (-0.19 to -0.01, p=.026) | -0.09 (-0.17 to -0.02, p=.017) |
| Z_HR_delta | [-1.6,2.6] | Mean ± SD | 0.0 ± 1.0 | -0.04 (-0.12 to 0.03, p=.257) | -0.02 (-0.11 to 0.07, p=.709) |  |
| ZNegative_T2_T1 | [-3.6,2.7] | Mean ± SD | 0.0 ± 1.0 | 0.00 (-0.08 to 0.08, p=.934) | 0.04 (-0.04 to 0.13, p=.293) |  |
|  | | | | | | |

**Comment 1.2:** Computational modeling

(a) The authors used the inequity between proposer and recipient as a crucial variable in their primary fMRI analysis. This approach is logical, as the inequity between the two is a reliable indicator of the injustice they are examining. However, this inequity term is not included in equation (1). Even if this exclusion is the result of model selection, it is difficult to believe that this model accurately represents injustice. Please clarify that inequity term has also an important role in modeling.
(b) It seems the authors employed RSS for model selection (equation (3)). As RSS doesn't compensate for the number of parameters, it can overfit data and select the model with the maximum number of parameters. The authors need to reanalyze the data using 10-fold cross-validation.

**Response：**

Thank you for providing your suggestions. Our model incorporates the concept of inequity, where “X1-50” and “X2-50” represent the unfairness towards others. Instead of directly calculating the difference between X1 and X2 in our formula, we opted for a separate approach in our experimental study. Specifically, we asked participants to punish X1 or help X2 individually, aiming to discern the extent of X1 receiving more and X2 receiving less. The combination of these two aspects is represented by X1-X2. To accomplish this, we utilized a fairness threshold of 50:50, dividing the subjective effects of the two parts and subsequently summing them up. This method ensures that the model parameters are not redundant and effectively predict participants’ behavior.

In our study, we employed the RSS method to estimate the model parameters, which is commonly used by previous studies (Stallen et al., 2018). This method is appropriate when participants' choices are continuous variables, and the dataset is relatively small. However, to compare different models while considering the number of parameters, we utilized the AIC (Akaike information criterion). For instance, in our study, both models 2 and 4 consisted of two parameters, whereas model 5 had four parameters. Through AIC comparison, we determined that model 4 was the optimal choice. It is important to note that RSS does not always favor models with a greater number of parameters.

To further assess the performance of the selected model, we conducted a 10-fold cross-validation. This involved comparing the simulated responses (the predicted amount of punishment or help) with the observed responses (the actual amount of punishment or help) to compute the cross-validated prediction accuracy. The accuracy rates ranged from 0.66 to 0.75, indicating the model’s capability to predict participants’ behavior.

Reviewer #3 shares the same opinion about computational modeling. Firstly, he/she note that “inequity is included in the full model (equation 1) because the payoffs of both players are included in the utility such that a higher inequity between the two players prompts the third-party to punish the transgressor more harshly and help the victim more”. Secondly, he/she note that “the authors used RSS to fit the model parameters but AIC to compare the models which takes care of the number of parameters”. Hopefully the above explanation will help you resolve some of your confusions.

**Comment 1.3:**

fMRI analysis

(a) The authors inconsistently switched their correction criteria in the fMRI analysis. They used a small volume correction for the amygdala but whole brain corrections for many cortical areas. A similar problem is apparent in ACC, PCC, and rTPJ, described on page 9. Even if this is due to the authors hypothesizing the amygdala's involvement in inequity processing, previous studies have also reported that dorsal and ventral striatum and many cortical areas, including the insula, DLPFC, ACC, PCC, and IFG, are involved in the processing of inequity. Please apply correction criteria systematically for the fMRI analysis and also include these areas for consideration.

**Response：**

Thank you for providing your valuable suggestions. In the original manuscript, we acknowledged that there were two correction strategies used, which may give an impression of using unsystematic correction criteria outlined in the guidelines. We have now used a more systematically stringent thresholding criteria, with a height threshold of p < 0.001 and an extent threshold of p < 0.05 corrected for multiple comparisons on the whole brain level according to suprathreshold cluster-size approach based on Monte-Carlo simulations. Given our a priori hypotheses on regions of interest in the amygdala and insula, we applied an additional procedure with small volume correlation for these ROIs.

First, we would like to clarify that we prioritized the more rigorous whole-brain correction approach and ensured that we reported whole-brain corrected results for each analysis, including those presented in Supplementary Materials Table S2-S6. We believe that by adopting this approach, we maintained a higher level of methodological rigor and accuracy in our study. However, we also aimed to explore specific brain regions that have consistently shown involvement in previous studies, even though they did not meet the threshold for whole-brain correction in our study. Therefore, we conducted additional analyses using small-volume correction (SVC), which was applied after the correction for multiple comparisons across the whole brain. The purpose of this correction was to improve the comparability of our findings with prior studies.

In the first level of our GLM analysis, we examined the aspect of “distributional inequity: |Proposer-Recipient|,” and in the second level, we investigated the differences in brain processing of this inequity between the stress and control groups. When selecting the regions of interest (ROIs), we prioritized brain regions that are relevant to both inequity processing and stress, including the Anterior cingulate cortex (ACC), amygdala and insula. Our results revealed a significant finding only in the amygdala, as reported in the main manuscript (Table S3). However, we acknowledge that this approach may introduce subjectivity, as it disregarded other potentially relevant regions associated with equity. Therefore, we further examined additional regions implicated in inequity processing in previous studies, such as the anterior insula (AI), putamen, ventrolateral prefrontal cortex (vlPFC), dorsal anterior cingulate cortex (dACC), dorsomedial prefrontal cortex (dmPFC), and dorsolateral prefrontal cortex (dlPFC). The coordinates for these regions were obtained from a previous meta-analysis by Feng et al. (2015). We defined the ROIs using a sphere with a diameter of 10 mm. The coordinates for these regions are as follows:

- Left dorsal ACC: [-4, 16, 48]
- Right dorsal ACC: [8, 22, 40]
- Right AI: [38, 20, 0]
- Left AI: [-30, 24, 2]
- Right putamen: [22, 12, 2]
- Left putamen: [-22, 12, 2]
- Right vlPFC: [34, 21, -18]
- Left vlPFC: [-44, 24, -8]
- Right dlPFC (middle frontal gyrus/sfg): [40, 36, 26]
- Left dlPFC (middle frontal gyrus): [-30, 38, 30]
- Right dmPFC: [8, 60, 16]
- Left rostral ACC/medial PFC: [-12, 46, 4]

Feng, C., Luo, Y. J., & Krueger, F. (2015). Neural signatures of fairness-related normative decision making in the ultimatum game: a coordinate-based meta-analysis. *Human brain mapping*, *36*(2), 591–602. https://doi.org/10.1002/hbm.22649

Consequently, none of these brain regions yielded significant results in the “Stress-Control”/ “Control-Stress”/ “Stress Conjunction Control” conditions after correcting for multiple comparisons (SVC corrected *P*_FWEs_ > 0.05, see Table R 1.3). This suggests that stress may not significantly interfere with an individual’s cognitive processing of unfairness per se, but it may affect their emotional responses when processing unfair events. Regarding this new part of the attempt, we have added a note in the Note section of TableS3.

**Table R 1.3: Neural response for increasing distributional inequity |Proposer-Recipient | in decision stage.**

|  |  | MNI Coordinates | | | T score | voxels | P_uncorr_ |
| --- | --- | --- | --- | --- | --- | --- | --- |
| Brain region and contrast | Side | X | Y | Z |  |  |  |
| **Control > Stress** | | | | | | |  |
| Left dorsal ACC | L | -12 | 18 | 48 | 1.19 | 149 | 0.113 |
| Right dorsal ACC | R | 12 | 26 | 38 | 1.80 | 211 | 0.038 |
| Right AI | R | 42 | 24 | -8 | 2.00 | 132 | 0.025 |
| Left AI | L | -22 | 26 | -2 | 1.80 | 276 | 0.038 |
| Right putamen | R | 18 | 20 | 0 | 1.41 | 127 | 0.079 |
| Right vlPFC | R | 28 | 26 | -18 | 2.95 | 316 | 0.002 |
| Left vlPFC | L | -52 | 24 | -6 | 1.65 | 418 | 0.051 |
| Right dlPFC | R | 48 | 34 | 30 | 1.42 | 291 | 0.077 |
| Left dlPFC | L | -24 | 38 | 22 | 0.26 | 8 | 0.329 |
| Right dmPFC | R | 14 | 56 | 22 | 1.68 | 231 | 0.049 |
| **Stress > Control** | | | | | | |  |
| Left dorsal ACC | L | -10 | 12 | 42 | 1.93 | 365 | 0.030 |
| Right dorsal ACC | R | 4 | 18 | 36 | 2.00 | 304 | 0.025 |
| Right AI | R | 42 | 12 | 0 | 2.74 | 383 | 0.004 |
| Left AI | L | -34 | 32 | -2 | 2.33 | 239 | 0.012 |
| Right putamen | R | 22 | 14 | 0 | 2.37 | 388 | 0.011 |
| Right vlPFC | R | 38 | 16 | -24 | 1.80 | 110 | 0.038 |
| Left vlPFC | L | -40 | 32 | -4 | 1.38 | 26 | 0.084 |
| Right dlPFC | R | 38 | 32 | 20 | 1.71 | 218 | 0.046 |
| Left dlPFC | L | -40 | 32 | -4 | 1.38 | 26 | 0.084 |
| Right dmPFC | R | 12 | 56 | 8 | 1.88 | 243 | 0.032 |
| **Conjunction** |  | | | | | |  |
| Left dorsal ACC | L | -18 | 44 | 8 | 1.30 | 23 | 0.095 |
| Right dorsal ACC | R | 16 | 22 | 34 | -0.49 | 4 | 0.496 |
| Right AI | R | 46 | 26 | 0 | -0.65 | 1 | 0.524 |
| Left AI | L | -22 | 18 | 2 | 2.04 | 21 | 0.023 |
| Right putamen | R | 22 | 18 | 2 | 1.59 | 189 | 0.057 |
| Right vlPFC | R | 40 | 24 | -24 | 1.94 | 164 | 0.029 |
| Left vlPFC | R | -44 | 24 | -18 | 1.83 | 108 | 0.036 |
| Right dlPFC | R | 36 | 28 | 22 | -1.31 | 515 | 0.594 |
| Left dlPFC | L | -22 | 18 | 2 | 2.04 | 21 | 0.023 |
| Right dmPFC | R | 2 | 60 | 24 | 1.83 | 140 | 0.036 |

In addition, taking your suggestion of “A similar problem is apparent in ACC, PCC, and rTPJ, described on page 9.”, in the revised manuscript, we have separated the whole-brain correction results from the SVC results, and similarly we have considered Amygdala and insula here in addition to ACC, still in view of their frequent reporting in stress-related studies. However, after conducting the analysis, we found that only the ACC region remained significant (a height threshold P < 0.001, an extent threshold P_FWE_ < 0.05 SVC corrected). We have made the necessary corrections in the new manuscript to accurately reflect these results. Thank you for bringing this to our attention, and we appreciate your contribution to improving our study.

***Materials and Methods***

**General linear model.** We regressed the fMRI time series into three general linear models (GLMs) to investigate how acute stress affected the brain’s decision circuitry. We looked for neural activity associated with inequity in the decision stage, i.e., the degree of inequity between the proposer and recipient (GLM_ Inequity) with the first GLM. We also checked the stress manipulation effect. In the second GLM (GLM_ Choice), we aimed to recognize brain regions with behavior associated with punishment and help choices under the unfair condition. The third GLM (GLM_ Utility) sought to identify brain regions coding utility and severity preference between punishment and help during the transfer stage. For the main contrasts, the individual voxel threshold was set to P < 0.001. We performed whole-brain corrections for multiple comparisons at the cluster level (P _FWE_ < 0.05). *Additionally, we conducted supplementary analyses to explore brain regions that have consistently emerged as relevant in previous studies and are aligned with our research design. These supplementary analyses involved small-volume correction (SVC), which was applied after the whole-brain multiple comparisons correction. The purpose of this correction was to enhance the comparability of our results with those of prior studies. Specifically, when examining the effects of stress, we performed small-volume correction on stress-related brain regions, including the amygdala and insula. Similarly, when investigating the processing of “inequity,” we applied small-volume correction to brain regions known to be involved in this process. The threshold for small-volume correction was set at P < 0.001 for individual voxels, with a cluster level of significance at P _FWE_ < 0.05.* *We defined the anatomical regions of interest (ROIs) for the bilateral amygdala, anterior cingulate cortex and insula using the SPM Wake Forest University (WFU) Pickatlas toolbox (www.ansir.wfubmc.edu, version 3.0)* (Tzourio-Mazoyer et al., 2002)*. The anatomical coordinates for the “inequity”-related areas were obtained from a previous meta-analysis. We used a sphere with a diameter of 8 mm as the size criterion for the ROIs, and the coordinates for these regions are provided in Table S9.* (page17, line 610)

***Results***

***Stress increases amygdala and prefrontal network integration in third-party intervention responses to inequality events in the decision phase.*** *To investigate the overall effect of acute stress on brain systems involved in the third-party intervention game (TPIG), we conducted a GLM with a parametric design*(Büchel et al., 1998) *to identify brain regions coding “Inequity” on the decision phase and the overall effect induced by stress. We initially conducted a whole-brain correction and observed increased activation in the right TPJ and right cerebellum in the stress group compared to the control group (****Table S2****, voxel level threshold P _uncorrected_ < 0.001; cluster level threshold P _FWE_ < 0.05). To further focus on specific brain regions related to acute stress, we applied a small-volume correction. This analysis revealed that the stress group exhibited higher activation in the left amygdala and bilateral insula compared to the control group (****c****, initial threshold P < 0.001; extent threshold of P _FWE_ < 0.05). These results confirm earlier findings indicating the essential role of the* *emotional salience network in the acute stress response, indicating the validity of CPT-induced changes in brain functioning*(Hermans et al., 2014)*. Critically, we found that the activity in the rTPJ, VLPFC, and DLPFC was positively correlated with distributional inequity between the proposer and the recipient for both the stress and control groups (Table S3, voxel level threshold P _uncorrected_ < 0.001; cluster level threshold P _FWE_ < 0.05, whole-brain corrected). These findings support the meta-analytical result on the neural processing of inequity by Feng et al. (2015)*(Feng et al., 2015)*. To further investigate other regions of interest (ROIs) related to inequity, we performed additional small-volume correction. Our results revealed a significantly higher correlation between the degree of distributional inequity and the activity in the right amygdala among control individuals compared to stressed individuals (****Fig 3A & Table S3****).* (page8, line 228)

***Acute stress alters brain functional connectivity and latent computations in the transfer phase.*** *To investigate the neurocomputational mechanisms of how acute stress alters functional brain systems involved in third-party intervention responses to inequality events, we first conducted a parametric modulation analysis with trial-by-trial subjective values (the “Utility” in the computational model) as a parametric modulator during the transfer phase. This analysis identified that the vmPFC, PCC, and several other regions were critical for the integrated subjective value of the transfer magnitude by maximizing utility for both stressed and control groups (****Fig 4B****;* ***Table S6****, height threshold P < 0.001, extent threshold P _FWE_ < 0.05 whole-brain corrected). However, after applying correction for multiple comparisons, no significant differences were found in brain regions associated with subjective value representation between the stress and control groups.*

*Motivated by the behavioral effect on the stress-induced decrease in prosocial punishment in the transfer phase, we investigated the neurocomputational correlates of punishment severity bias (punishment relative to help, α-β) during the transfer stage. Through whole-brain multiple regression analysis, we discovered that punishment severity bias exhibited a stronger correlation with the activity of the PCC and rTPJ in stressed versus control participants (Table S6, voxel level threshold P _uncorrected_ < 0.001; cluster level threshold P _FWE_ < 0.05, whole brain corrected). Additionally, small-volume correction revealed that punishment severity bias displayed a stronger correlation with the activity of the ACC in stressed participants compared to control participants (Table S6, voxel level threshold P _uncorrected_ < 0.001; cluster level threshold P _FWE_ < 0.05, SVC corrected). However, the amygdala and insula, which are highly associated with stress, did not exhibit differences between the stress and control groups in this context. To further analyze the aforementioned notable brain regions, we discovered a positive correlation between punishment bias and activity in the ACC, PCC, and rTPJ in the stress group, while the control group showed negative correlations (****Fig 4A)****.*  (page9, line 286)

(b) On page 9, the authors stated, "Interestingly, after correcting for multiple comparisons, we did not observe consistent differences in brain regions in subjective value representation between the stress and control groups." Why is this interesting rather than being not significant?

**Response：**

Thank you for your suggestion. Here are the modifications made in the new manuscript:

*However, after applying correction for multiple comparisons, no significant differences were found in brain regions associated with subjective value representation between the stress and control groups.* (page9, line 293)

**Response to Reviewer #2**

Thank you sending me this interesting manuscript. The manuscript investigated the effect of acute stress on third party punishment. The authors conclude that acute stress:
-decreased the third party's willingness to punish the violator
-decreased the severity of the punishment
-increased the willingness to help the victim

I think the findings are interesting and make a solid contribution to the field. I would be happy to read a revised version of the manuscript. Here are my comments and suggestions which I feel should be addressed before publication.

**Response：** We are glad that you commend our paper “make a solid contribution to the field.” Your comments and suggestions were very helpful to improve our manuscript. We have rewritten and reinterpreted our findings on the basis of your suggestions. Below we describe in detail how we responded to all key points mentioned in your report (text written in blue color).

**Comment 2.1:**

Abstract
1. the use of the term "intervention severity bias" in the abstract is rather ambiguous. Either explain clearly what this is or remove it from the abstract

**Response：** Thank you for your suggestion. In the revised manuscript, we have chosen to replace the term “intervention severity preference” with “preference of justice recovery.” This modification is based on the understanding that the original term referred to the preference between two significant parameters, α and β, in our computational model. These parameters represent the severity of punishment (reflecting one’s aversion to witnessing harm inflicted on others) and helpfulness (reflecting one’s aversion to seeing others harmed), respectively. Since the participant's role in this study involves self-costly justice recovery, we believe that the revised term better captures the essence of the concept.

**ABSTRACT**

*Computational modeling revealed a shift in preference of justice recovery from punishment the offender toward help the victim under stress. This finding is consistent with the increased dorsolateral prefrontal engagement observed with higher amygdala activity and greater connectivity with the ventromedial prefrontal cortex in the stress group. A brain connectivity theory-of-mind network predicted stress-induced justice recovery in punishment. (page2, line 38)*

**Comment 2.2:**

Abstract
2. "Computational modeling revealed a shift in intervention severity bias from punishment toward help under stress. This finding is consistent with the increased dorsolateral prefrontal engagement observed with higher amygdala activity and greater connectivity with the ventromedial prefrontal cortex." Is this in the stress group? If so, please clarify.

**Response：**

Thank you for bringing this expression error to our attention. Indeed, we have found “greater connectivity between the amygdala and vmpfc” in the stress group, and we have made the necessary correction in the new manuscript:

**ABSTRACT**

*Computational modeling revealed a shift in preference of justice recovery from punishment the offender toward help the victim under stress. This finding is consistent with the increased dorsolateral prefrontal engagement observed with higher amygdala activity and greater connectivity with the ventromedial prefrontal cortex in the stress group. A brain connectivity theory-of-mind network predicted stress-induced justice recovery in punishment. (page2, line 38)*

**Comment 2.3:**

Intro
3. When reviewing the literature on third party punishment under stress, the authors do not acknowledge an important recent meta-analysis which did not find consistent effects of acute stress on third party punishment:

-Does Stress Make Us More—or Less—Prosocial? A Systematic Review and Meta-Analysis of the Effects of Acute Stress on Prosocial Behaviours Using Economic Games: https://doi.org/10.1016/j.neubiorev.2022.104905

**Response：**

After carefully reviewing the above-mentioned meta-analysis, we were pleasantly surprised to find that a great work had accomplished what we had long been planning to do. Over the past decade, numerous studies have examined the impact of acute stress on pro-social behaviors using the economic game paradigm, but the results have been inconsistent. This meta-analysis provides a comprehensive overview by summarizing 23 studies on pro-social behavior in economic decision-making. The findings indicate that acute stress does not influence pro-social decisions or costly punishment in various economic games such as the Dictator Game, Trust Game, Ultimatum Game, or Prisoner’s Dilemma. This work holds significant value as it suggests new avenues for future research in this field, such as exploring how stress affects other forms of prosociality and considering the role of effort in influencing prosocial behavior.

In our current study, we define pro-social framing in terms of justice restoration. Maintaining justice allows us to establish, promote, and enforce long-term pro-social social norms. However, an important detail to note is that in our research paradigm, regardless of whether participants choose to punish violators or help victims in the process of justice restoration, they assume the role of the “third party.” This role is distinct from the responder’s role in the Ultimatum Game (Player B). To illustrate this distinction, we can compare second-party punishments (e.g., Ultimatum Game, Player B) with third-party punishments (e.g., Ultimatum Game, Player B) and gain a better understanding of third-party punishments.

Firstly, we concur that social norms can be reinforced through both second-party punishment (SPP) and third-party punishment (TPP), and both forms are considered prosocial, and in the majority of legal systems and societies, moral norms are mainly upheld through the implementation of third-party punishment. Norm violations directly impact the economic payoff of second parties, whereas third parties are not directly affected by such violations(Fehr & Fischbacher, 2004). The majority of studies indicate that third-party punishment is considered prosocial due to its deterrent effect on selfish behavior and the self-cost involved(Boyd et al., 2003, 2010; Fehr et al., 2002). Moreover, the emotional motivations underlying second-party punishment (SPP) and third-party punishment (TPP) may vary. Second-party punishment, which can be viewed as a self-defense mechanism, may be influenced by the affected individual’s direct self-interest(Civai et al., 2012) and a desire for revenge (Strobel et al., 2011). Research has indicated that third parties tend to prefer equitable outcomes, whereas second parties tend to favor unequal but personally advantageous outcomes(Civai et al., 2012). Third-party punishment systems, which take into account the harm suffered by victims and evaluate the motives of wrongdoers, seem to possess a more executive nature compared to affective second-party punishment systems (Krueger & Hoffman, 2016).

Hence, the absence of significant effects of acute stress on costly punishment, as observed in the meta-analysis titled “Does Stress Make Us More—or Less—Prosocial? A Systematic Review and Meta-Analysis of the Effects of Acute Stress on Prosocial Behaviors Using Economic Games,” does not conclusively dismiss the potential influence of acute stress on third-party punishment, considering the potential divergence in underlying motivations for SPP and TPP. However, we firmly believe that the “Hypotheses and literature review” section should incorporate a more comprehensive range of citations, including both supportive and conflicting evidence. In response to this concern, we have made revisions to the introduction section of the manuscript. The following are the revised sections:

**INTRODUCTION**

*Decision-making under stress can follow a “tend and befriend” pattern, where decision behavior can be both adaptive and altruistic, promoting survival and well-being at both individual and group levels*(Taylor, 2006)*. Nevertheless, it is important to note that the “tend and befriend” pattern does not always hold true, as some studies have demonstrated a decrease in prosocial preferences under acute stress* (Forbes et al., 2023; Oriel FeldmanHall1 et al., 2015; Schulreich et al., 2022)*, while others have found no significant effects in economic games (according to a recent meta-analysis)* (Nitschke et al., 2022)*.* **(Page 2, Line 54)**

*Third-party punishment and help are both examples of altruistic behavior, and they share a common neuronal mechanism. However, they also involve distinct processes* (Stallen et al., 2018)*. When facing injustice, both third-party responses correlate with the activation of fairness violations, e.g., the anterior insula (AI), anterior cingulate cortex (ACC), dorsolateral prefrontal cortex (DLPFC), striatal reward circuitry and emotional regulation systems, including the amygdala and ventromedial prefrontal cortex (vmPFC)*(Stallen et al., 2018; Xie et al., 2022)*. Enhanced ventral striatal activity is further associated with punishing an offender instead of helping a victim* (Stallen et al., 2018)*. On the other hand, the mentalizing network, including the temporoparietal junction (TPJ), precuneus, and mid-temporal regions, is more strongly associated with an individual's preference for helping rather than punishing* (Civai et al., 2019)*. In other circumstances, activity in the participants’ right dorsolateral prefrontal cortex (rDLPFC) increased more after experiencing advantageous unfairness and engaging in help instead of punishment, whereas those who punish more after experiencing disadvantageous unfairness demonstrated significantly decreased activity in the rDLPFC*(Xie et al., 2022)*. It is important to note that the DLPFC is not only involved in mentalizing* (Tusche et al., 2016) *but also plays a critical role in context-dependent cognitive control* (Tusche & Hutcherson, 2018)*. Although studies have demonstrated that stressed individuals have greater altruistic* (Takahashi et al., 2007; von Dawans et al., 2012)*, cooperative, and other-oriented tendencies*(Nickels et al., 2017)*, the neurocognitive mechanisms underlying the trade-off between third-party help and punishment under acute stress remain unclear.* **(Page 2, Line 76)**

**Comment 2.4:**

Additionally on p. 2 they write:
"Acute stress can lead to increased activation of "theory of mind" 72 (ToM) brain regions (Tomova et al., 2017), which is fundamental for becoming more other-oriented with a preference for providing help (FeldmanHall et al., 2015)"

Again please see the meta-analysis above and also consider more recent findings which suggest that stress does not always lead to increased prosocial behaviour:

-Acute stress reduces effortful prosocial behaviour: https://doi.org/10.7554/eLife.87271.1
-Altruism under Stress: Cortisol Negatively Predicts Charitable Giving and Neural Value Representations Depending on Mentalizing Capacity: https://doi.org/10.1523/JNEUROSCI.1870-21.2022

**Response：**

Thank you for your thoughtful comment. In a recent meta-analysis, Nitschke et al. successfully combined multiple economic game paradigms to demonstrate that acute stress does not always result in increased prosocial behavior. However, it's important to note that this finding might be specific to economic games, and it does not necessarily negate the impact of acute stress on other measures of prosocial behavior, such as moral judgments, compassionate behaviors, or different economic games like third-party punishment and help(Nitschke et al., 2022).

A recent study by Forbes et al. exemplifies this point. They utilized an effort-based task that better approximates real-life prosocial behavior and found that participants in the stress group, compared to controls, chose to exert more physical effort for self-benefitting rewards at a low level of effort (Effort level 2). However, no significant difference was observed at other effort levels (Effort level 1, 3, 4, 5). Additionally, the adverse effects of stress on prosocial effort were only observed in more selfish participants rather than prosocial individuals (Forbes et al., 2023).

In Schulreich et al.'s study, neural activity associated with charitable giving under stress was assessed. They discovered that while stress manipulation itself did not influence charitable giving, increases in the stress hormone cortisol were associated with reductions in donations among participants with high mentalizing capacity but not among low mentalizers. Furthermore, Schulreich et al.'s study also revealed that the stress group exhibited significantly higher charitable giving compared to the control group during the pre-manipulation phase of stress. In fact, the stress group displayed higher charitable giving both before and after the stress manipulation, relative to the control group. Therefore, it is not entirely conclusive that Schulreich et al.'s study provides strong evidence supporting the notion that stress reduces prosocial behavior (Schulreich et al., 2022).

Additionally, when considering the moderating effects of other factors, Schulreich et al.'s study seems to have opposite conclusions to Forbes et al.'s study. Given that Forbes et al. found decreases in prosocial effort only in more selfish participants, whereas Schulreich et al. found reductions in donations in participants with high mentalizing capacity. This may indicate that mentalizing capacity and prosocial behavior are not entirely synonymous, as mentalizing capacity may represent more of an "other regarding" ability to attribute mental states (e.g., beliefs, desires, intentions) to others. In Schulreich et al.'s study, they found that activations in the right dorsolateral prefrontal cortex (DLPFC) were less predictive of post-manipulation donations in high mentalizers with increased cortisol. Other regions associated with the Theory-of-Mind (ToM) network, such as the DMPFC, temporoparietal junction (TPJ), and middle temporal gyrus (MTG), did not show significant predictive results. Overall, we agree with your previous suggestion to consider both positive and opposing evidence more comprehensively. In the revised manuscript, we have made several statements in the Introduction section to address this concern.

**INTRODUCTION**

*Decision-making under stress can follow a “tend and befriend” pattern, where decision behavior can be both adaptive and altruistic, promoting survival and well-being at both individual and group levels*(Taylor, 2006)*. Nevertheless, it is important to note that the “tend and befriend” pattern does not always hold true, as some studies have demonstrated a decrease in prosocial preferences under acute stress* (Forbes et al., 2023; Oriel FeldmanHall1 et al., 2015; Schulreich et al., 2022)*, while others have found no significant effects in economic games (according to a recent meta-analysis)* (Nitschke et al., 2022)*.* **(Page 2, Line 54)**

**Comment 2.5:**

p. 3 "we hypothesized that people under stress would prefer helping when third-party punishment and helping are in conflict because punishment is more executive control-dependent." Why should punishment be more dependent on executive control than helping?

There are many studies showing that prosocial behaviour requires cognitive control (e.g. inhibiting selfish responses). The dlPFC and parietal regions are consistently activated by prosocial tasks e.g. see this meta-analysis:
-Neural signatures of prosocial behaviors: https://doi.org/10.1016/j.neubiorev.2020.07.006
Why should this be different under stress?

**Response：**

We would like to clarify that our previous statements did not refute the association between prosocial behavior and cognitive control. Rather, we tend to favor the notion that third-party punishment relies more on cognitive control capacity compared to third-party help. This perspective does not contradict the findings of the meta-analysis conducted by (Bellucci et al., 2020) .

Bellucci et al.'s meta-analysis revealed that brain regions consistently activated during prosocial behaviors partially overlap with mentalizing regions (dorsal posterior cingulate cortex) and empathy-related areas (middle cingulate cortex). These findings support the hypothesis, based on psychological research, that socio-cognitive and empathic abilities are central to prosociality. However, the authors also found that the ventromedial prefrontal cortex (vmPFC) and particularly the dorsolateral prefrontal cortex (dlPFC) were preferentially recruited during prosocial acts. Importantly, the dlPFC was the only brain region preferentially engaged by prosocial behaviors and not activated during empathy or mentalizing tasks. This suggests that prosocial behaviors require the involvement of other crucial processes, such as the valuation and planning of prosocial actions. Upon examining the experiments contributing to the activation of the dlPFC in this meta-analysis, we observed that at least half of the evidence came from studies employing the prisoner's dilemma (PD) paradigm, such as Watanabe et al. (2014), Emonds et al. (2012), Telzer et al., and other studies utilizing reinforcement learning paradigms like Stanley D. A. (2016). Costly punishment in the prisoner's dilemma and reinforcement learning both necessitate specific neural computations, such as beliefs about the character and reputation of the interacting partner, thus requiring activation of the dlPFC. In another related study by Hu et al., they investigated the relationship between third-party punishment, third-party help, and empathy concern. They observed that distinct networks were involved in the two processes when compared to separate control conditions. Helping activated the bilateral striatum and right lateral prefrontal cortex (lPFC), while punishment activated the bilateral striatum, left lPFC, and ventral medial prefrontal cortex (vmPFC). However, their study did not directly compare third-party punishment and third-party help. Nonetheless, previous research has demonstrated that third-party punishment recruits the dlPFC, which is crucial for executive functioning and integrating multiple information streams for appropriate decision-making (Buckholtz et al., 2008; Sanfey et al., 2003; Zinchenko, 2019). Similarly, in our study, we do not deny that third-party help also involves executive control functions, but we are more inclined to agree that third-party punishment relies more on the executive control functions of the dlPFC.

**Comment 2.6:**

Results
4. The interaction between Group x Fairness x Intervention is not significant (p>.10). I would not label this as a 'trend effect'. Also, the decision to conduct the analysis only on the 'extreme' unfairness condition is not really justified. Why was this done?

**Response：**

Thank you for bringing up this concern. We have made modifications to address the "trend effect" in the revised manuscript.

*The analysis conducted revealed a lack of statistical significance in the Group × Fairness × Intervention interaction (F (3, 150) = 2.811, P = 0.108, η_p_^2^ = 0.040). (page5, line 167)*

We focused more on extreme unfair conditions, on one hand, as we mentioned in the supplementary file, we compared the punishment, help and selfish choices, as a function of the fair condition (50:50, 60:40, 70:30, 80:20, 90:10). As expected, we found that as the degree of unfairness increased, the proportion of punishment and help behavior increased, and the proportion of selfish behavior decreased correspondingly and this effect was similar in the stress and control group (**Fig S1A&B**, stress main effect of choice rate: F (1, 50) = 0.71, P = 0.40, *η*_p_^2^ = 0.014; stress main effect of contribution: F (1, 50) = 1.04, P = 0.31, *η*_p_^2^ = 0.020).” In addition, as mentioned in the original text, "A further exploratory analysis revealed that acute stress affects decision-making only in extremely unfair conditions (i.e., 80:20 and 90:10 trials), with an increase in third-party decisions to help (P< 0.05 in 80:20 and 90:10 conditions) but a decrease in third-party decisions to punish (P < 0.05 in 80:20 and 90:10 conditions) in the stress group compared with the control group (Fig 2A)." We discovered the effects of acute stress specifically in the context of extreme unfairness, where there was a higher proportion of decisions to help compared to punish. The comparison between these two conditions was the main focus of our study, which is why we concentrated more on this aspect in the supplementary file.

On the other hand, we have also taken into account other unfair conditions in our study. These conditions are reflected in:

**DATA ANALYSIS**

**“**Furthermore, we combined 6:4 and 7:3 as relatively unfair conditions (RUF), 80:20 and 90:10 as extreme unfair conditions (EUF), and 50:50 as the fair condition (F). The clustering criterion was established based on the findings of a previous meta-analysis (Oosterbeek et al., 2004). Repeated measurement ANOVA was used in two conditions (RUF and EUF).**”** *(page5, line 167)*

**SUPPLEMENTARY FILE**

We also regressed the fMRI time series into a general linear model (GLM_ Choice2) to investigate how acute stress affected the brain’s decision circuitry. In the GLM (GLM_ Choice2), we aimed to recognize brain regions whose behavior was associated with punishment and help choice under all the unfair conditions (60:40~90:10). We defined the following two onset regressors of interest: (i) onset of the punishment choice of all unfair trials, (ii) onset of the help choice of all unfair trials. We also defined the following uninterested regressors: (iii) onset of the punishment transfer of all unfair trials, (iv) onset of the help transfer of all unfair trials, (v) onset of all the choices of fair trials (5:5, for punishment, help and keep choice), (vi) onset of all the transfers of fair trials (5:5, for punishment, help and keep choice). The GLM additionally included six movement regressors of no interest, three for translational movements (x, y, z) and three for rotation movements (pitch, roll, yaw). All regressors were convolved with the canonical hemodynamic response function. Individual contrast images (for “Punishment”, “Help”) were transferred to a second-level analysis using 2 (group) by 2 (Choice) mixed factorial analysis of variance (ANOVA). More specifically, several participants were excluded in the second level because coefficients for the parameters could not be estimated when participants never, or only one time, choosing help or punishment per functional run. To this end, the group contrast was computed with 48 participants (stress group: 25; and control group:23), By comparing trials in which punishment is selected vs trials in which help is selected between stress and control group under all unfair conditions, we found that stress induced a higher activity in right DLPFC, PCC and right Thalamus when select punishment choices relative to help choices (Figure S3, initial threshold set at P uncorrected < 0.001, whole-brain cluster corrected at P FWE < 0.05).

Furthermore, our computational model is also simulated based on data from all fair conditions, and the subsequent analyses in the study are focused on all fair conditions as well.

**Comment 2.7:**

Discussion
5. Could the findings be explained by differences in framing effects under acute stress (e.g. Pabst et al., 2013). Punishing always involved deducting money (loss frame) whereas being prosocial was always adding money (gain frame). How can the author be sure this is a social effect of acute stress rather than a framing effect?

**Response：** Thank you for raising this possibility. We have reviewed relevant studies on framing effects, but we ultimately found it somewhat implausible to attribute our findings solely to framing effects.

Framing effects refer to how information is presented or framed and its impact on decision-making. Positive or gain-oriented framing tends to make individuals more risk-averse, leading them to prefer options offering certainty. Conversely, negative or loss-oriented framing tends to make individuals more risk-seeking, prompting them to favor options that offer a chance to avoid losses. In the study conducted by Pabst et al. (2013), they investigated decision-making in both gain and loss domains using a modified version of the Game of Dice Task (GDT). However, our study did not involve risk-based decision-making, and we did not manipulate framing-related information in the instructions. Although participants may have defined their own frames when making choices, defining a clear frame in our design was challenging. For instance, from the participants’ perspective (in our design, both help and punishment are costly), punishment could signify a deduction of money (a loss frame), while help could also involve a cost, resulting in a reduction of money. However, from the collective perspective of the three individuals (A+B+C), help would mean adding money (a gain frame) since participant C incurs a cost to benefit participant B threefold. Consequently, it was difficult to unambiguously establish a loss or gain frame in our study. Moreover, if we assume that punishment represents a loss frame and help represents a gain frame, participants’ behavior should not be influenced by the level of fairness. However, our data clearly indicate that fairness is a significant variable influencing decision-making, and our computational model suggests that participants' decisions are influenced by their aversion to inequality, leaning towards a social effect.

Therefore, although framing effects could be a potential explanation, we believe that other factors, such as fairness and inequality aversion, play a more substantial role in our study.

**Comment 2.8:**

6. p. 10 "a growing body of studies has demonstrated that stressed individuals have greater altruistic…" See comments/references in the introduction. The effects of acute stress on altruism/generosity are far from conclusive, please acknowledge this.

**Response：** Thank you for your comment. As mentioned earlier, Nitschke et al. successfully combined multiple economic game paradigms to demonstrate that acute stress does not always result in increased prosocial behavior. However, it's important to note that this finding might be specific to economic games, and it does not necessarily negate the impact of acute stress on other measures of prosocial behavior, such as moral judgments, compassionate behaviors, or different economic games like third-party punishment and help(Nitschke et al., 2022). However, our previous statements may have been too definitive. We have made some linguistic modifications in the new manuscript, as follows:

*Decision-making under stress can follow a “tend and befriend” pattern, where decision behavior can be both adaptive and altruistic, promoting survival and well-being at both individual and group levels*(Taylor, 2006)*. Nevertheless, it is important to note that the “tend and befriend” pattern does not always hold true, as some studies have demonstrated a decrease in prosocial preferences under acute stress* (Forbes et al., 2023; Oriel FeldmanHall1 et al., 2015; Schulreich et al., 2022)*, while others have found no significant effects in economic games (according to a recent meta-analysis)* (Nitschke et al., 2022)*.* **(Page 2, Line 54)**

**Comment 2.9:**

7. p. 11 "helping behavior under stress is more intuitive and straightforward and becomes a more adaptive and habitual decision with relatively lower cognitive and computational expenditure." / "punishment is no longer an optimal strategy in dealing with other-regarding injustice events since it requires more cognitive and mentalizing resource involvement" (again see comment in the introduction). I feel the authors are just describing the results again but do not really explain why helping is less cognitively demanding under stress and/or why punishment is more cognitively demanding?

**Response：** Thank you for pointing out this question and the direction you provided. Our previous discussion on this topic was indeed relatively weak. Regarding the cognition demand of punishment, we partially addressed it in response #2.5. Previous research has shown that third-party punishment involves the recruitment of the dlPFC, which is crucial for executive functioning and integrating multiple information streams for appropriate decision-making (Buckholtz et al., 2008; Sanfey et al., 2003; Zinchenko, 2019).

In the new manuscript, we have expanded the discussion on this topic.

**DISCUSSION**

*Besides, we found that stress increased amygdala-vmPFC functional connectivity under the punishment options, previous research on third-party punishment has also discovered a strengthening of amygdala connectivity with lateral prefrontal regions that are involved in punishment decision-making*(Treadway et al., 2014) *. Amygdala-vmPFC connectivity has previously been found to play a crucial role in reward- and value-based decision-making* (Gangopadhyay et al., 2020; Maier et al., 2015)*, as well as in the generation and regulation of negative emotions* (Wager et al., 2008)*, and various aspects of social cognition, such as facial emotion recognition, theory-of-mind ability, and processing self-relevant information* (Hiser & Koenigs, 2018)*. Our findings suggest that acute stress may lead to a reduction in general activation in the amygdala’s response to inequity. However, when it comes to complex third-party punishment decision, stress may induce hypercoupling of brain networks involved in value computations, regulation of aversive emotions, and mentalizing.* **(Page 13, Line 399)**

*We also found that stressed individuals demonstrated stronger rACC, rPCC, and rTPJ activities when computing the relative severity of punishment against help than those in the control group. The ACC has been shown to balance the motivational conflict between the immediate emotional reaction and maximize profit in the decision-making context* (Botvinick, 2007; Sanfey et al., 2003)*.* *Moreover, we found that acute stress plays a moderating role in the direction of value computation of the rACC and rPCC to the relative preference between punishment and help and subsequently affects the transfer level of punishment relative to help. Specifically, the stressed participants needed to use more cognitive rACC and rPCC resources for a larger punishment severity bias and to impose a greater punishment contribution. In contrast, individuals in the control group only need to invest a low level of rACC and rPCC resource consumption in deciding how severely to punish.* *These findings suggest that punishment, rather than helping, is a more deliberative response pattern. Under stress, people direct greater cognitive resources for value-based computation and emotion regulation when making punishment decisions.* **(Page 13, Line 413)**

**Response to Reviewer #3**

Summary of the paper:
The main questions of this paper are how acute stress changes the third-party's intervention policies (here punishing the perpetrator or helping the victim) after observing injustice, and what neural mechanisms underly these intervention decisions under stress.

To induce acute stress in half of the participants, the authors used the cold-pressor test, and used measures of salivary cortisol concentration, subjective ratings of negative affect (PANAS) and heart rate to show the stress induction was successful. The authors designed a third-party intervention paradigm where the participants observed a dictator game between players A (dictator) and B (recipient), and they had to decide whether and how much to punish the dictator or help the recipient, using their endowment; the participants could also decide to keep their endowment and not intervene at all. In each trial, they varied how unfair player A's offer was (dividing 100 monetary units in 5:5, 6:4, 7:3, 8:2 and 9:1 portions) and quantified the proportion of each of the three possible intervention decisions in response (keeping the MUs, transferring their Mus to reduce player A's payoff, transferring their MUs to increase player B's payoff), as well as the amount of MUs transferred to A and B.

The authors find that acute stress increases the rate of helping decisions and decreases punishment, only in the extremely unfair conditions (8:2 and 9:1 division). They developed four computational models and showed that the best fitting model is one that combines self-payoffs and other-regarding inequality aversion to obtain the utility of each decision. They showed that the difference between two model latent parameters alpha (how averse a person is to observe someone else hurt others) and beta (how averse a person is to observe someone else being hurt), conceptualizing the model estimates of punishment bias, correlates with both punishment bias in choice rates and amount of contributions; moreover, the model punishment bias is modulated by the stress condition, such that punishment bias is lower in the stress compared to the control condition. They also investigated the neural correlates of such third-party intervention decisions and how stress alters neural activity.

All in all, this paper is a useful addition to the literature, however, the authors need to revise the manuscript before publication. Below are some suggestions for how to improve the paper.

**Response：** Thank you for providing such a well-organized and clear summary of our study. We greatly appreciate your positive evaluation and recognition of our work. We carefully reviewed your suggestions, paying close attention to each point, and found them all to be highly constructive. Your suggestions have significantly enhanced various aspects of our research, including the hypotheses and literature review, task design and description, computational model, neuroimaging, and discussion and writing. Moving forward, we will address your suggestions one by one and make the necessary improvements. It is possible that some of the areas still have room for further enhancement after the revisions. We sincerely thank you once again for taking the time to review and provide corrections to our research.

**Comment 3.1: Hypotheses and literature review:**
The authors need to make sure they are providing a more thorough review of the existing literature in motivating their hypotheses, and more explicitly state alternative hypotheses and review the supporting evidence. Also, the effect of using different paradigms for inducing stress needs to be discussed in more detail in the Discussion section. The authors reviewed evidence and base their hypotheses on the findings that stress increases trust and altruistic behavior (e.g., von Dawans et al 2012a). However, they did not cite the opposing evidence, for example in FeldmanHall et al. 2015, finding that stress indeed decreases trust towards others. Indeed, the stressor test used in FeldmanHall paper is CPT (the one also used in the current manuscript), whereas the von Dawans paper used the Trier Social Stress Test which induces psychosocial stress by requiring individual to undergo social evaluation. As written in FeldmanHall et al (2015) "CPT eliminates the putatively confounding psychosocial effects on social behavior … inducing non-social stress does not increase trusting behavior but rather attenuates trusting behavior". Therefore, the authors should have made the opposite prediction for the effect of acute stress on decisions of punishment versus helping, given that they are using CPT.

**Response：**

Thank you for your wonderful suggestion. We acknowledge that there are studies with inconsistent findings that we did not mention initially. Our primary focus in the original text was on exploring the trade-off between third-party helping and punishment, both of which fall within the realm of altruistic behavior. In our study design, we included a selfish (keeping) condition as a shared non-altruistic control for both third-party helping and punishment. Upon reconsideration of your point, we agree that it would be more objective to first discuss the relationship between acute stress and altruistic behavior, given the lack of complete consistency in research findings. Subsequently, we can address the trade-off between third-party helping and punishment within the framework of altruism. To address this concern, we have included a clarification in the revised manuscript's Introduction section.

Regarding the hypothesis section, we concur with FeldmanHall et al. (2015) on the domain-specific effects of acute stress induction. They proposed that when considering social cognition and behavior, the influence of prosocial behavior resulting from acute stress is uncertain due to the potential influence of the social nature of the stressor used in the study (i.e., being evaluated while speaking publicly) rather than the neurobiological stress response itself. They highlighted the importance of using the CPT to eliminate the confounding psychosocial effects on social behavior. We align with this perspective and, within the framework of social cognition and behavior, employed the CPT as our stress induction method. Moreover, during the manuscript revision process, we explored which components of stress (cortisol/heart rate/negative affect) drive changes in social behavior under stress. Our findings indicate that an increase in cortisol is responsible for the observed increase in helping behavior and the decrease in punishment (see Supplementary Materials Table S7 and S8).

However, while we support FeldmanHall et al.'s (2015) viewpoint, we cannot solely rely on their research to formulate an alternative hypothesis favoring punishment over help. We revisited their study and indeed found that “those who were not under stress exhibited no differences between the amount of money gambled and the amount of money entrusted to partners. In comparison, stressed subjects gambled more money but entrusted less money to partners.” However, their study did not find that stressed individuals exhibited reduced trust in a social context (trust game) compared to the control condition, as this result is most directly relevant to our current study. Furthermore, recent studies utilizing the MIST or TSST stress induction methods have found reduced prosocial behavior in a social context, and these effects were influenced by individual differences such as selfishness(Forbes et al., 2023) or mentalizing capacity(Schulreich et al., 2022). In conclusion, we support FeldmanHall et al.'s (2015) perspective when discussing the differences between their results and von Dawans et al.'s (2012) study from the standpoint of stress induction methods. Their study employed a non-social CPT induction, and the main highlight of their findings is the interaction between the social context (trust game vs. gamble) and stress. We maintain our current hypothesis for several reasons. Firstly, we incorporate the dual-process theory of decision-making along with the well-established finding that acute stress impairs executive functions. Moreover, we take into account the distinction between third-party punishment and help, as mentioned in the previous manuscript. Secondly, the research conducted by von Dawans et al. (2012 and 2019) also offers supporting evidence. In their 2012 study, participants who underwent acute social stress induced by the TSST demonstrated significantly greater prosocial behavior, including trust, trustworthiness, and sharing, compared to participants in a control condition who did not experience the TSST. In von Dawans et al.'s 2019 study, they expanded upon the 2012 task by introducing a punishment game (we concur that “reject” behavior in the punishment game falls under prosocial behavior). They observed that acute stress exposure heightened prosocial trustworthiness and sharing, while also leading to lower levels of punishment among women without contraception in the stress condition. Essentially, in a study similar to the prosocial behavior we are investigating, they found both an increase in trust induced by acute stress and a decrease in punishment. Consequently, their findings lend some support to our current research hypothesis. However, we acknowledge that the method used to induce stress may have influenced the results partly. We will address this point in the revised manuscript.

**INTRODUCTION:**

*Decision-making under stress can follow a “tend and befriend” pattern, where decision behavior can be both adaptive and altruistic, promoting survival and well-being at both individual and group levels*(Taylor, 2006)*. Nevertheless, it is important to note that the “tend and befriend” pattern does not always hold true, as some studies have demonstrated a decrease in prosocial preferences under acute stress* (Forbes et al., 2023; Oriel FeldmanHall1 et al., 2015; Schulreich et al., 2022)*, while others have found no significant effects in economic games (according to a recent meta-analysis)* (Nitschke et al., 2022)*. Stress can profoundly impact psychological, neurophysiological, and brain networks involved in third-party decision-making, thereby influencing the affective response, motivation, and subjective value assigned to the decisions* (Phelps et al., 2014; Ulrich-Lai & Herman, 2009)*.* **(Page 2, Line 54)**

*Third-party punishment and help are both examples of altruistic behavior, and they share a common neuronal mechanism. However, they also involve distinct processes* (Stallen et al., 2018)*. When facing injustice, both third-party responses correlate with the activation of fairness violations, e.g., the anterior insula (AI), anterior cingulate cortex (ACC), dorsolateral prefrontal cortex (DLPFC), striatal reward circuitry and emotional regulation systems, including the amygdala and ventromedial prefrontal cortex (vmPFC)*(Stallen et al., 2018; Xie et al., 2022)*. Enhanced ventral striatal activity is further associated with punishing an offender instead of helping a victim* (Stallen et al., 2018)*. On the other hand, the mentalizing network, including the temporoparietal junction (TPJ), precuneus, and mid-temporal regions, is more strongly associated with an individual's preference for helping rather than punishing* (Civai et al., 2019)*. In other circumstances, activity in the participants’ right dorsolateral prefrontal cortex (rDLPFC) increased more after experiencing advantageous unfairness and engaging in help instead of punishment, whereas those who punish more after experiencing disadvantageous unfairness demonstrated significantly decreased activity in the rDLPFC*(Xie et al., 2022)*. It is important to note that the DLPFC is not only involved in mentalizing* (Tusche et al., 2016) *but also plays a critical role in context-dependent cognitive control* (Tusche & Hutcherson, 2018)*. Although studies have demonstrated that stressed individuals have greater altruistic* (Takahashi et al., 2007; von Dawans et al., 2012)*, cooperative, and other-oriented tendencies*(Nickels et al., 2017)*, the neurocognitive mechanisms underlying the trade-off between third-party help and punishment under acute stress remain unclear.* **(Page 2, Line 76)**

*In addition to the trade-off of third-party help and punishment at a behavioral level, it remains unclear whether the two candidate decisions involve differential neurocomputational processes; for instance, whether and with what severity to intervene in an injustice event under uncontrollable stress*(Hu et al., 2015; Stallen et al., 2018)*. The computational model allows us to decipher signals that are not expressed by the behavioral indicators, e.g., in value utility calculations, people rely only on intuition and habit in the allocation of some choices (e.g., helping), while others (e.g., punishing) are considered more carefully and deliberately. We can identify specific neural circuits linked to specific decision variables and processes by combining economic computational tools and neuroimaging approaches. Therefore, it is critical to investigate the neurocomputational bases of third-party intervention under acute stress by integrating economic paradigms, computational tools, and neuroimaging approaches. Based on the biphasic-reciprocal model of the stress-induced transition from deliberate to intuitive under stress, we hypothesized that people under stress would prefer helping when third-party punishment and helping are in conflict because helping victims is considered a more habitual response in stressful situations, which aligns with “tend and befriend” concept due to its social approach property* (Buchanan & Preston, 2014; Taylor, 2006)*. However, previous research has demonstrated that third-party punishment involves the activation of the DLPFC, which plays a crucial role in executive functioning and the integration of multiple information streams for appropriate decision-making* (Buckholtz et al., 2008; Sanfey et al., 2003; Zinchenko, 2019)*. We therefore expected that acute stress would decrease behaviors that require adequate cognitive control while increasing behaviors that recruit habitual and reflexive regulation.* **(Page 3, Line 94)**

**DISCUSSION:**

*These findings suggest that when individuals are under stress, they tend to exhibit a more deliberative response pattern towards punishment rather than helping. It appears that people allocate greater cognitive resources towards value-based computation when making decisions related to punishment. It is important to note that the sample collected for this study consisted only of males, so the results may be more applicable to men than women. Furthermore, given that a physical stressor was employed in this study, it would be intriguing to investigate the impact of a social stressor, such as the Social Stress Test (TSST), on decision preferences. We are inclined to believe that a social stressor may engage the empathy and mentalizing networks to a greater extent when making decisions related to helping. Previous research has indeed shown that acute stress can enhance prosocial behavior by intensifying the sharing of others’ emotions* (Tomova et al., 2017). (Page 13, Line 422)

**Comment 3.2: Task design and description.**
There are missing important details about the task design in the main text.
**Comment 3.2.1**

- It is not clear whether the participants saw both punish and help screens in the "transfer phase". It seems they do, and it's not clear whether they were instructed to choose zero MUs for the decision they had not made (e.g., choose zero MUs on the help screen if they chose "punish" in the "decision phase").

**Response：**

Participants could observe both the amount they punished and the amount they helped during the "transfer" phase. They were not explicitly instructed to choose zero monetary units (MUs) for decisions they did not make. If participants chose "punish" during the decision phase, they would prioritize the amount allocated for punishment during the transfer phase. Afterward, they would decide how much of the remaining amount they want to allocate towards helping.

**Comment 3.2.2**

- In the task description, it is not clear whether they vary the level of unfairness during the player A-B interaction. This is only mentioned for the first time in the results section.

**Response：**

During the decision phase, participants were not explicitly informed about the fairness level of the allocation between Player A and Player B. However, in reality, we designed five different fairness levels: 5:5, 6:4, 7:3, 8:2, and 9:1. The actual offers shown to the participants varied between 1% and 2%. For example, offers such as 91/9, 92/8, 90/10, 88/12, and 89/11 all belong to the 9:1 fairness level. These different ratio offers were randomly presented to the participants.

**Comment 3.2.3**

- Also, it is not clear whether they allow A's MUs to get negative, e.g., if the third-party deducts more than what A has from the dictator game stage.
**Response：**

In the instructions, we do not explicitly inform participants that the monetary units (MUs) of participant A can be reduced to a negative value. This is because, according to the cover story provided to participants, A and B are portrayed as previous volunteers in our study. As a result, the offers exchanged between A and B affect their variable income, rather than their base income. Therefore, participants understand that A’s MU can be reduced to a minimum of 0, however, A can get their base income. However, during the decision-making process, participants do not have real-time visibility of A's current final amount after their adjustments. Therefore, the punishment process requires participants to calculate the final amount themselves. Consequently, it is possible for some participants, driven by strong punishment motives or due to calculation errors, to reduce A's monetary units (MUs) to 0.

**Comment 3.2.4**

- The order of the help and punish screens do not seem to be counterbalanced, but this is not explicitly mentioned.

**Response：**

The punishment and help transfer stages are not counterbalanced. The order of these stages depends on the option chosen by the participants during the decision stage. If participants choose the "punish" option, the transfer stage will first present the punishment and then the help. Conversely, if they choose the "help" option, the transfer stage will first present the help and then the punishment. In the case of choosing the "keep" option, both transfer stages will involve keeping the amounts, and participants are explicitly informed that selecting "keep" will not lead to any changes in A's and B's monetary units (MU) or their own MU.

**Comment 3.2.5**
Beside clarifying the task description, this last point is particularly important to be considered. The authors need to explain this design choice and also discuss how it might have affected their results. Based on how the computational model is formulated later, it becomes clear that the authors assume the help and punish decisions happen in parallel before seeing each decision page, however, it is possible that the participants engage in a serial decision making process according to the task design. Considering a serial decision making process, if the participants can decide to transfer non-zero MUs in both punish and help screens in the transfer phase, the starting endowment for the punish and help are not equal. In other words, they always have equal or less MUs to begin with for the help versus the punish decisions.

**Response:** Thank you for raising this interesting point. As mentioned in my previous response (response 3.2.4), the order of the punishment and help in the transfer stage is determined by the participants’ choices during the decision stage. If a participant selects "punish" during the decision stage, the punishment stage will be presented first, followed by the help stage. Conversely, if they choose "help," the help stage will precede the punishment stage. However, it's important to note that the decision made during the decision stage only determines the order of presentation in the transfer stage. Participants retain the ability to determine the magnitude of punishment or help they provide.

Based on the comments above, here are some modifications to the description of the task design:

***Third-party Intervention Task.***

*In the decision stage, participants were presented with three options: transferring MUs to decrease player A's MUs, transferring MUs to increase player B's MUs, or keeping all the MUs for themselves. If participants chose to reduce player A's money during the decision stage, they could first decide on the amount of MUs to transfer from their own initial 50 MUs and deduct that amount from player A's MUs in increments of 5 MUs in the first transfer stage. Following that, participants could further decide on the amount of MUs to transfer from their remaining MUs to compensate player B in the second transfer stage. On the contrary, if they chose "increase player B" during the decision stage, they could first determine the amount of MUs to transfer to player B during the first transfer stage and then proceed to choose a reduction in player A's MUs during the second transfer stage (see Fig 1E). It is important to note that the MUs transferred by participants will be applied to player A or player B at a triple ratio. For example, if they transfer 5 MUs to increase player B, player B will actually receive an increase of 15 MUs. Each run of the experiment consisted of 30 preprogrammed trials, and a total of 3 runs were conducted. The offers presented to the participants were specifically crafted, with average offer ratios ranging from 90/10 to 50/50. However, the actual offers shown during the experiment varied between 1% and 2%, for example, 91/9 and 88/12. The composition of fairness levels in the offers was not disclosed to the participants. Participants were aware that their decisions would not cause player A's income to become negative, as they were informed that their choices only affected the "floating" portion of the income for players A and B, not the "base" portion. (page 14, line 477)*

**Comment 3.3: Computational model**
**Comment 3.3.1**

Please clarify whether Fig 2G-H report model predictions on training or held-out/cross validation data.

**Response：**

Figures 2G-H display the model predictions on the training data. Since we didn't previously separate the data into training and validation sets, this time we have managed to evaluate the model's accuracy using 10-fold cross-validation. The results showed an average value of 0.66 across the 10 folds.

**Comment 3.3.2**
The computational model in line 512 is a model of parallel decision making for the transfer phase. Two questions that need to be answered by the authors.
- First, can the same parameters fit to each individual's transfer phase data be used to predict their decisions between punish/help/keep in the "decision phase"? Second, in the transfer phase, the participants see the punish and help screens serially and not in parallel; whereas the model makes such decisions in parallel. How would considering a serial decision-making process affect the results of using the model to assess punishment bias, and later in using the model parameters in neural data analysis?

**Response：**In response to your question, we conducted an additional analysis to examine the relationship between the parameters α and β (two parameters in the win model) and the rates of help and punish in the decision stage (In each trial, the probabilities of punish, help, and keep sum up to 1). The results of our analysis revealed that α is positively correlated with the proportion of punish and negatively correlated with the proportion of help. Conversely, β is negatively correlated with the proportion of punish and positively correlated with the proportion of help. These findings are presented in Table 3.3.2.1. Hence, these results demonstrate a strong correlation between the model parameters in the transfer stage and the choices made in the decision stage.

**Table 3.3.2.1 The correlations between model parameters and decision rates.**

| Dependent Variable | | Unstandardized Coefficients | | Standardized Coefficients | t | p |
| --- | --- | --- | --- | --- | --- | --- |
|  |  | B | Std. Error | Beta |  |  |
| Help rate (UF) | (Constant) | .291 | .103 |  | 2.824 | .007 |
|  | β | .445 | .097 | .475 | 4.569 | .000 |
|  | α | -.336 | .078 | -.448 | -4.310 | .000 |
| Punish rate (UF) | (Constant) | .409 | .095 |  | 4.301 | .000 |
|  | β | -.370 | .090 | -.387 | -4.114 | .000 |
|  | α | .463 | .072 | .605 | 6.435 | .000 |
| Help rate (EU) | (Constant) | .363 | .118 |  | 3.066 | .004 |
|  | β | .524 | .112 | .445 | 4.678 | .000 |
|  | α | -.522 | .090 | -.553 | -5.821 | .000 |
| Punish rate (EU) | (Constant) | .598 | .119 |  | 5.031 | .000 |
|  | β | -.531 | .112 | -.444 | -4.720 | .000 |
|  | α | .537 | .090 | .561 | 5.968 | .000 |
| Help rate (All) | (Constant) | .220 | .084 |  | 2.615 | .012 |
|  | β | .374 | .080 | .494 | 4.688 | .000 |
|  | α | -.250 | .064 | -.413 | -3.924 | .000 |
| Punish rate (All) | (Constant) | .332 | .076 |  | 4.362 | .000 |
|  | β | -.302 | .072 | -.389 | -4.192 | .000 |
|  | α | .380 | .058 | .612 | 6.590 | .000 |

Note: UF, A: B 6:4,7:3,8:2,9:1; EU, A: B 8:2,9:1; All, A: B 5:5, 6:4,7:3,8:2,9:1.

Regarding the second question, our modeling process does not take into account the sequentially of the transfer stage. The order of whether to punish or help first in the transfer stage is determined by the participants' choices in the decision stage. If an individual chooses to punish first, we assume that they have a preference for punishment. Consequently, we would expect their punishment amount in the first stage (transfer One) to be higher than the punishment amount in the second stage (transfer Two). Similarly, if they choose to help in the decision stage, we predict that their helping amount in the first transfer stage would exceed the helping amount in the second transfer stage. Our data confirms this observation. We compared the punishment amounts between the transfer One and transfer Two stages for each participant and found a significantly higher punishment amount in transfer One. The same pattern was evident for helping amounts, as depicted in the table 3.3.2.2. Therefore, in our modeling process, we do not explicitly consider the sequence of decisions, but rather focus on the relative preference between punishment and helping. Nevertheless, we acknowledge that modeling a potential serial decision-making process would be wise to explore in future studies.

**Table 3.3.2.2 The cost differences between two transfer stage.**

| **Paired Samples Test** | | | | | | | | | |
| --- | --- | --- | --- | --- | --- | --- | --- | --- | --- |
|  | | Paired Differences | | | | | t | df | p |
|  |  | Mean | Std. Deviation | Std. Error Mean | 95% Confidence Interval of the Difference | |  |  |  |
|  |  |  |  |  | Lower | Upper |  |  |  |
| Pair 1 | Punish cost (Transfer One) – Punish cost (Transfer Two) | 10.26893 | 8.47295 | 1.17499 | 7.91004 | 12.62782 | 8.740 | 51 | .000 |
| Pair 2 | Help cost (Transfer One) – Help cost (Transfer Two) | -6.46769 | 10.99618 | 1.52490 | -9.52904 | -3.40633 | -4.241 | 51 | .000 |

**Comment 3.3.3**

Two corrections:
- The pi_t terms in equation 4 line 530 should be replaced by 50, because utility should be equal to pi_t which is 50 minus the contributions for punishment and helping. Moreover, the S_p and S_h terms should not be multiplied by 3. Just as written correctly in line 542.

**Response：**Thank you for highlighting these two points. We have carefully considered your input and incorporated the necessary revisions into our updated manuscript.

***Model 1: Baseline model***

The baseline model proposes a rational assumption that participants made decisions only by maximizing their self-payoff. That is, the absence of inequality aversion hypotheses and participant choice not to help and punish instead would be an optimized option. The utility function was as follows:

$U_{i(t)}=50-S_{i\left( t \right)}^{p}- S_{i\left( t \right)}^{h} (4)$

**Comment 3.3.4**
- I'm not sure I understand the sentence in line 548-549: "participants used the same but distinct psychological mechanisms when determining whether to help or punish others."

**Response：**Here, what we originally intended to express participants show variations in their sensitivity to inequity (captured by parameters α and β), but they exhibit consistent sensitivity to the differences between themselves and others. This is why our final model does not include an additional parameter to differentiate their own cost/gain relative to others. However, we have taken your suggestion into account and simulated a new model (model6), which will be described in more detail in the subsequent questions.

**Comment 3.3.5**
There are three classes of models that worth considering as well, especially if the authors want to use the abstract latent parameters in the model to dissociate the underlying neural mechanisms of bias towards punishment versus helping decisions (see the last paragraph in the neuroimaging section of this review).
- In the "other-regarding inequity aversion", participants are modeled as being motivated to punish dictators who keep an unfair amount of MUs for themselves, and the participant's sensitivity to this unfairness is formalized as alpha term. The participant gets utility by decreasing this amount of inequity by punishing the dictator. The more the punishment balances out this inequity the more the participant will gain utility, until the punishment completely balances the inequity out; however, participants will not lose utility if they are being overly harsh towards the dictator. From the literature on punishment, we do know that people prefer proportional punishment, that is they are sensitive to unfairness and do not want to be too lenient, but they also do not want to overly harm the transgressor. The authors did not consider such class of models that allow for loss of utility due to being overly harsh. Indeed, in their current model, people tend not to be overly harsh because being overly harsh is tied to their own selfish gains; the harsher they punish the more money they will lose themselves.

**Response：**

Thank you for your constructive suggestion. In response to this potential issue, we made the following attempts:

Firstly, we calculated the proportion of participants who punished dictators with negative values (see Figure 3.3.5). Among the 52 participants, we found that 27 participants did not punish any dictators with negative values in any trial. While there were instances where certain participants applied negative punishments, the average occurrence of such cases was only 2.01%. Therefore, we conclude that the decision-making strategies of the majority of participants are not excessively severe, and even the most punitive participants did not exceed 20% trials. This finding aligns with recent research on distributive justice, which combines an experimental paradigm with fMRI and integrated neurocomputational modeling. The results of this study indicate that individuals consider three motives (inequality aversion, harm aversion, and rank reversal aversion) when engaging in justice redistribution. Thus, people generally adhere to the "do-no-harm" principle and tend to avoid rectifying injustice at the expense of harming others or reversing social rankings(Li et al., 2022). This example effectively illustrates the fundamental conflicts in distributive justice, which often fuel intense debates in real-life scenarios, such as finding ways to increase taxation on wealthy individuals while safeguarding the interests of the entire population and maintaining social order. Furthermore, it is worth noting that third-party punishment can indeed act as a deterrent. Nevertheless, as evidenced by another real-life example, when legal judgments are passed against criminals, leniency is frequently sought instead of resorting to extreme forms of punishment.

Secondly, in Model 2 (Self-regarding inequality aversion model), we accounted for the factors of excessive punishment and excessive help. We introduced the concept of “advantageous inequality” (parameter ‘guilt’) to capture this behavior, which represents participants’ consideration of situations where they possess more resources than the transgressor or receiver. Our analysis revealed that the average value of advantageous inequality in the dataset was 4.53, whereas the average value of disadvantageous inequality (reflecting situations where participants have fewer resources than the transgressor or receiver) was 34.32. This indicates that participants rarely take ‘guilt’ into account, thus making instances of excessive punishment infrequent. However, through model comparisons, we found that although Model 2 (Self-regarding inequality aversion model) incorporates this aspect, it is weaker compared to our final model.

Based on the attempts made above and the research findings of previous researchers, we believe that punishing dictators to a negative value is not a common decision-making strategy.

**Figure 3.3.5. Proportion of Punished Dictators with Negative Values.**

**Comment 3.3.6**

- The inequity aversion terms in the full model also formulate inequity between player A and player B. In addition to these terms, the participants might be averse to inequity between themselves and player A/B as well. The authors have not considered such class of models (although they considered self-regarding inequity, but not a combination of self and other-regarding inequity).

**Response：**

We have previously considered this model, but after conducting some basic calculations on the data, we decided to pass on it as we found it unsuitable for fitting our data. We computed the correlation between AI, DI, and Difference_X1-X2 in Model 2, where Difference_X1-X2 is defined as the maximum of dictator(finally) - receiver(finally) and 0. We found a high correlation between these variables (as described in Table 3.3.6). In such a case, attempting to fit the model would result in parameter redundancy because of the high correlation between DI and Difference_X1-X2, as well as AI and Difference_X1-X2. It would be difficult to separate different psychological processes within a single model. Therefore, we abandoned the possibility of using this model.

**Table 3.3.6 The correlation between DI, AI, Difference of X1 and X2.**

|  | r | [95% CI] | p |
| --- | --- | --- | --- |
| DI-AI | -0.22 | [-0.47, 0.05] | 0.114 |
| DI-Diff (x1-x2) | 0.62 | [ 0.42, 0.76] | <.001 *** |
| AI- Diff (x1-x2) | 0.30 | [ 0.03, 0.53] | 0.029 * |

We did attempt to fit Model 5:

$$\begin{aligned} U_{i\left( t \right)}\left( S_{i\left( t \right)}^{p},S_{i\left( t \right)}^{h} | \alpha_{i},\beta_{i},\mathrm{envy}_{i},\mathrm{guilt}_{i} \right)= \pi_{i\left( t \right)}-\left\{ \max\left[ \alpha_{i}\cdot\left( x_{1\left( t \right)}-50 \right)-3S_{i\left( t \right)}^{p}, 0 \right]-\min\left[ \beta_{i}\cdot\left( x_{2\left( t \right)}-50 \right)+3S_{i\left( t \right)}^{h},0 \right] \right\} \\ -\mathrm{envy}_{i} \cdot\mathrm{DI}_{i} -\mathrm{guilt}_{i}\cdot\mathrm{AI}_{i} \#\left( \right) \end{aligned}$$

$$\mathrm{DI}_{t}=\max\left[ x_{1\left( t \right)}-3S_{i\left( t \right)}^{p}-\pi_{i\left( t \right)},0 \right]+max\left[ x_{2\left( t \right)}+3S_{i\left( t \right)}^{h}-\pi_{i\left( t \right)},0 \right] \left( \right)$$

$$\mathrm{AI}_{t}=\max\left[ \pi_{i\left( t \right)}{-(x}_{1\left( t \right)}-3S_{i\left( t \right)}^{p}),0 \right]+max\left[ {\pi_{i\left( t \right)}-(x}_{2\left( t \right)}+3S_{i\left( t \right)}^{h}),0 \right] \left( \right)$$

In this model, we take into account both the unfairness between X1 and X2 and the unfairness between the participant (X3) and the other two. However, the results of the model comparison still indicate that the optimal model is the one we used before (Table 3.3.7).

**Comment 3.3.7**
- Consider adding a constant multiplier to part in equation (2), line 512, in order to allow for differential weighting of the self-regarding utility compared to the other-regarding utility terms. Because individuals might not only vary in terms of how sensitive they are to inequity (captured in alpha and beta terms), but also how strongly they weigh their own cost/gain relative to others'. Not including a necessary constant in the model would create a bias in estimating the true values of aversion to inequity which are the main target of model fitting in this paper.

**Response：**

Thank you for your suggestion. We have taken your input into consideration and developed a new model, referred to as Model 6. In this model, we incorporate the idea that participants exhibit variations not only in their sensitivity to inequity (represented by alpha and beta terms), but also in the extent to which they prioritize their own costs and gains compared to those of others. To capture this trade-off, we have introduced a parameter λ. The formulation of Model 6 is as follows:

$$\begin{aligned} U_{i\left( t \right)}\left( S_{i\left( t \right)}^{p},S_{i\left( t \right)}^{h} | \alpha_{i},\beta_{i},\lambda_{i} \right)=\lambda_{i} \cdot\pi_{i\left( t \right)}-(1-\lambda_{i})\left\{ \max\left[ \alpha_{i}\cdot\left( x_{1\left( t \right)}-50 \right)-3S_{i\left( t \right)}^{p}, 0 \right]-\min\left[ \beta_{i}\cdot\left( x_{2\left( t \right)}-50 \right)+3S_{i\left( t \right)}^{h},0 \right] \right\} \\ \#\left( \right) \end{aligned}$$

However, the results of the model comparison still indicate that the optimal model is the one we used before (Table 3.3.7).

**Table 3.3.7 The overview of model comparisons.**

| **Model** | **Description of the models** | **AIC** |
| --- | --- | --- |
| Model 1 | Baseline model | 477.807038483339 |
| Model 2 | Self-regarding inequality aversion model | 392.351260533234 |
| Model 3 | Other-regarding inequality aversion model with shared parameters for estimating the severity of punishment and help. | 381.167204053846 |
| Model 4 | Other-regarding inequality aversion model | **361.187649224120** |
| Model 5 | Self-Other-regarding inequality aversion model 1 | 397.381785153329 |
| Model 6 | Self-Other-regarding inequality aversion model 2 | 374.434780052375 |

Based on the above, after considering your suggestions and conducting further analysis, we have explored additional potential models to fit our data. However, as we hypothesized, the previous model we discussed remains the most suitable option. In the revised manuscript, we have included the results of these additional model attempts in the supplementary materials for comprehensive reporting.

**Comment 3.4: Neuroimaging**

**Comment 3.4.1**
The authors make a series of strong reverse inferences that need to be stated more cautiously and speculatively. For future work, reverse inferences can be strengthened by identifying functional regions using localizer tasks. Approximate locations in whole brain analyses are not specific enough to support the kinds of inferences made here.

**Response：**

Thank you for your suggestion. We have made certain revisions to the language and eliminated some overly speculative conclusions in the Discussion section.

***DISCUSSION***

*Besides, we found that stress increased amygdala-vmPFC functional connectivity under the punishment options, previous research on third-party punishment has also discovered a strengthening of amygdala connectivity with lateral prefrontal regions that are involved in punishment decision-making*(Treadway et al., 2014) *. Amygdala-vmPFC connectivity has previously been found to play a crucial role in reward- and value-based decision-making* (Gangopadhyay et al., 2020; Maier et al., 2015)*, as well as in the generation and regulation of negative emotions* (Wager et al., 2008)*, and various aspects of social cognition, such as facial emotion recognition, theory-of-mind ability, and processing self-relevant information* (Hiser & Koenigs, 2018)*. Our findings suggest that acute stress may lead to a reduction in general activation in the amygdala’s response to inequity. However, when it comes to complex third-party punishment decision, stress may induce hypercoupling of brain networks involved in value computations, regulation of aversive emotions, and mentalizing.*

(Page 12, Line 399)

*These findings suggest that when individuals are under stress, they tend to exhibit a more deliberative response pattern towards punishment rather than helping. It appears that people allocate greater cognitive resources towards value-based computation when making decisions related to punishment. It is important to note that the sample collected for this study consisted only of males, so the results may be more applicable to men than women. Furthermore, given that a physical stressor was employed in this study, it would be intriguing to investigate the impact of a social stressor, such as the Social Stress Test (TSST), on decision preferences. We are inclined to believe that a social stressor may engage the empathy and mentalizing networks to a greater extent when making decisions related to helping. Previous research has indeed shown that acute stress can enhance prosocial behavior by intensifying the sharing of others’ emotions* (Tomova et al., 2017) (Page 13, Line 422)

**Comment 3.4.2**
- Higher activation in amygdala, TPJ and bilateral insula in stress compared to control was used to conclude "role of emotional salience network in the acute stress response, indicating the validity of CPT-induced changes in brain functioning". However, they also find a higher correlation between right amygdala activity and the degree of distributional inequity in the control than the stress condition, which goes against their previous claim. So, it's really not as clear as stated by the authors what these regions are exactly doing in this task.

**Response：**

Thank you for pointing out this confusing point. “Higher activation in the amygdala, TPJ, and bilateral insula in stress compared to control” was a main effect of stress among all fair conditions. We conducted this analysis to check the performance of the CPT manipulation. Among the whole-brain correction, only the right TPJ and right cerebellum survived FWE correction. Additionally, we examined other regions of the salience network (Hermans et al., 2011, 2014), including the amygdala and insula, using small-volume correction (SVC) analysis. Since this is not the primary result of the study, we have presented it in the supplementary materials. In this revised version, we have provided a more detailed description of the GLM and analysis process for these results, which can be found in Table S2. In our findings, we observed a stronger correlation between right amygdala activity and the degree of distributional inequity in the control group compared to the stress condition, as indicated in the parametric design of the “GLM_Inequity.” This suggests that, as the degree of inequity increases, amygdala activation is relatively higher in the control group compared to the stress group. This finding aligns with previous evidence that suggests amygdala activity can predict the level of inequity aversion in prosocial individuals (Gao et al., 2018; Haruno & Frith, 2009; Yu et al., 2014). However, it is also possible that stress reduces the processing of negative emotions associated with unfair situations. In the revised manuscript, we have made more targeted modifications to the results and discussion sections, clarifying these two potentially confusing results.

**RESULTS**

*We initially conducted a whole-brain correction and observed increased activation in the* *right TPJ and right cerebellum in the stress group compared to the control group (****Table S2****,* *voxel level threshold P _uncorrected_ < 0.001; cluster level threshold P _FWE_ < 0.05). To further focus on specific brain regions related to acute stress, we applied a small-volume correction. This analysis revealed that the stress group exhibited higher activation in the left amygdala and bilateral insula compared to the control group (****c****, initial threshold P < 0.001; extent threshold of P _FWE_ < 0.05). These results confirm earlier findings indicating the essential role of the emotional salience network in the acute stress response, indicating the validity of CPT-induced changes in brain functioning*(Hermans et al., 2014)*. Critically, we found that the activity in the rTPJ, VLPFC, and DLPFC was positively correlated with distributional inequity between the proposer and the recipient for both the stress and control groups (Table S3, voxel level threshold P _uncorrected_ < 0.001; cluster level threshold P _FWE_ < 0.05, whole-brain corrected). These findings support the meta-analytical result on the neural processing of inequity by Feng et al. (2015)*(Feng et al., 2015)*. To further investigate other regions of interest (ROIs) related to inequity, we performed additional small-volume correction. Our results revealed a significantly higher correlation between the degree of distributional inequity and the activity in the right amygdala among control individuals compared to stressed individuals (****Fig 3A & Table S3****).* (Page 8, Line 232)

**DISCUSSION**

*The neuroimaging results demonstrate that acute stress leads to higher activity in the insula, amygdala, and TPJ, reflecting a hyperactive state of the emotional salience network, as observed in previous studies*(Hermans et al., 2014)*. Critically, we found that stress altered the preference for punishment and helping by redistributing brain activity associated with value calculation and mentalizing.* *These findings will be discussed in a framework of behavioral and neurocomputational mechanisms of how acute stress affects third parties’ decisions acting upon emotional salience, prefrontal control and mentalizing networks.*  (Page 11, Line 344)

*In conjunction with local activation, we observed that the activity in the rTPJ, VLPFC, and DLPFC was positively correlated with distributional inequity between the proposer and the recipient for both the stress and control groups, this corresponds to the early findings about inequity process*(Feng et al., 2015)*. Additionally, we discovered a stronger correlation between right amygdala activity and the degree of distributional inequity in the control group, in contrast to the stress condition. However, it is important to note that previous research has demonstrated the predictability of degree of inequity aversion from amygdala activity*(Gao et al., 2018; Haruno & Frith, 2009; Yu et al., 2014)*. Our results indicate that stress diminishes the processing of negative emotions associated with unfair situations. Besides, we found that stress increased amygdala-vmPFC functional connectivity under the punishment options, this result is consistent with previous studies that demonstrated that psychological stress and early life stress increased vmPFC functional connectivity with the amygdala*(Arnsten, 2015; Carmichael & Lockhart, 2018; Ginty et al., 2019; Sinha et al., 2016)*. Simultaneously, animal research and human studies have suggested that top-down regulation of the vmPFC to the amygdala is implicated in emotion regulation and extinction learning*(Burghy et al., 2013; Phelps & LeDoux, 2005)*. Overall, our findings suggest that acute stress may contribute to a decreased initial flexibility in the amygdala’s response to inequity. Additionally, participants experiencing stress tended to rely more on emotion regulation in the vmPFC to mitigate their aversion to inequity when making decisions regarding punishment.*  (Page 12, Line 397)

**Comment 3.4.3**
- In lines 261-263, the authors wrote "these results indicate that punishment, but not helping, might be a more intuitive and automated process, since we observed that the control group was more likely to choose the punishment option and exerted less cognitive effort to make punishment decisions". This is a huge reverse inference. Also, such inferences require a direct comparison of punishment and helping decisions, for example the contrast of punish>help or help>punish. Fig S3 shows such analysis, for brain regions that show an interaction between choice (punish/help) and group (stress/control). This analysis revealed thalamus, PRCC and rDLPFC; however, these brain areas do not strongly support the claim about intuitive processing of either helping or punishment decisions.

**Response：**

Thank you for raising your concerns here and in the Discussion section (Comment 3.5.3, Comment 3.5.4, Comment 3.5.5). We concur with your opinion that our current findings reveal an interaction between choice (punish/help) and group (stress/control) within the thalamus, rRCC, and rDLPFC. Moreover, we observed a heightened activation in the rDLPFC, rPCC, and rTPJ regions among individuals in the stress group when making punitive choices. However, we did not observe a direct effect of the help choice. Consequently, it's a reverse inference to chain that third party help is intuitive. In the revised manuscript, we have placed greater emphasis on the direct consequences of punishment and have provided a plausible explanation: We propose that third-party punishment under stress necessitates more deliberate consideration due to increased recruitment of cognitive control and value computation-related regions.

***DISCUSSION***

*Our results have provided a possible explanation, to some extent, that third-party punishment may require more deliberation compared to helping others. It entails greater cognitive control, and reliance on calculations. This thoughtful and pro-social choice is temporarily impeded under stressful conditions.*

*Broadly, people typically prefer to punish the offender than help the victim for justice restoration* (FeldmanHall et al., 2014; Stallen et al., 2018)*. Under stress, the previously observed considerate response underwent a significant transformation, and the effectiveness of punishment as a relatively desirable strategy for addressing instances of injustice involving others was diminished.* (Page 12 Line 384)

*Specifically, the stressed participants needed to use more cognitive rACC and rPCC resources for a larger punishment severity bias and to impose a greater punishment contribution. In contrast, individuals in the control group only need to invest a low level of rACC and rPCC resource consumption in deciding how severely to punish.* *These findings suggest that when individuals are under stress, they tend to exhibit a more deliberative response pattern towards punishment rather than helping. It appears that people allocate greater cognitive resources towards value-based computation when making decisions related to punishment.* (Page 13 Line 422)

**Comment 3.4.4**
The following points need to be addressed about the neural data analysis.
- In Fig 4A, the negative slope in the control condition seems to be driven by a single participant's data in the upper left corner of these plots. Please repeat the analyses excluding the outliers.

**Response：**

Thank you for bringing this to our attention. First, we would like to explain that the three brain regions in Fig4A are the ones that we found in the whole-brain multiple regression analysis that could pass the correction threshold, and the correlation plots in Fig4A, just for visualization and interpretive purposes, we extracted the β-values of these three brain regions. Therefore, we believe that the results of these three brain regions are still relatively reliable. Considering your concern about the outliers, we took a certain criterion (removing values other than the three standard deviations) to remove the outliers in the plots during visualization this time, and recalculated the correlation coefficients as follows:

Upon further examination, we have confirmed that in Fig4A, the values in the top left corner of the third graph exceeded three standard deviations. However, we feel that it is important to note that the top left corners of the first and second graphs did not contain values surpassing the three standard deviations threshold. Furthermore, our investigation revealed an additional outlier in the top right corner of the first graph, exceeding three standard deviations (as indicated in the table 3.4.4.1).

| **Table 3.4.4.1** Descriptive Statistics | | | | | | |
| --- | --- | --- | --- | --- | --- | --- |
|  | N | Range | Minimum | Maximum | Mean | Std. Deviation |
| α-β | 52 | 1.68 | -.89 | .79 | .0036 | .40681 |
| rTPJ_44_-60_40 | 52 | 15.29 | -3.41 | 11.88 | .9986 | 2.48432 |
| rPCC _2_-40_24 | 52 | 8.85 | -3.46 | 5.40 | .4784 | 1.74087 |
| rACC_2_36_16 | 52 | 11.43 | -5.04 | 6.39 | -.5085 | 1.86333 |
| Valid N (listwise) | 52 |  |  |  |  |  |

As a result, we have attempted to remove the outliers in the top right corner of the first graph and the top left corner of the third graph and subsequently reanalyzed the data：

After excluding the outlier in the top right corner of the first graph in Fig4A, a reanalysis was conducted, revealing a marginally significant positive correlation in the stress group (refer to Table 3.4.4.2).

| **Table 3.4.4.2 Correlations** | | | | |
| --- | --- | --- | --- | --- |
| group | | | α-β | ACC_2_36_16 |
| Control | α-β | Pearson Correlation | 1 | -.519^**^ |
|  |  | Sig. (2-tailed) |  | .008 |
|  |  | N | 25 | 25 |
|  | ACC_2_36_16 | Pearson Correlation | -.519^**^ | 1 |
|  |  | Sig. (2-tailed) | .008 |  |
|  |  | N | 25 | 25 |
| Stress | α-β | Pearson Correlation | 1 | .383 |
|  |  | Sig. (2-tailed) |  | .053 |
|  |  | N | 27 | 26 |
|  | ACC_2_36_16 | Pearson Correlation | .383 | 1 |
|  |  | Sig. (2-tailed) | .053 |  |
|  |  | N | 26 | 26 |
| **. Correlation is significant at the 0.01 level (2-tailed). | | | | |

After removing the outlier in the top left corner of the third graph in Fig4A, a reanalysis was conducted. The results revealed that the previously significant negative correlation observed in the control group was, in fact, attributable to the presence of the outlier (refer to Table 3.4.4.3).

| **Table 3.4.4.3 Correlations** | | | | |
| --- | --- | --- | --- | --- |
| group | | | α-β | rTPJ_44_-60_40 |
| Control | α-β | Pearson Correlation | 1 | -.175 |
|  |  | Sig. (2-tailed) |  | .414 |
|  |  | N | 25 | 24 |
|  | rTPJ_44_-60_40 | Pearson Correlation | -.175 | 1 |
|  |  | Sig. (2-tailed) | .414 |  |
|  |  | N | 24 | 24 |
| Stress | α-β | Pearson Correlation | 1 | .192 |
|  |  | Sig. (2-tailed) |  | .337 |
|  |  | N | 27 | 27 |
|  | rTPJ_44_-60_40 | Pearson Correlation | .192 | 1 |
|  |  | Sig. (2-tailed) | .337 |  |
|  |  | N | 27 | 27 |

We have made several updates in the revised manuscript, including the figures, result descriptions, and discussions pertaining to this particular outcome. Furthermore, in the subsequent analysis of the mediation effects, we have addressed the issue of outliers by removing them from the dataset and conducting a reanalysis.

**Comment 3.4.5**
- Please fix Fig 3A which shows the amygdala seed and connectivity with vmPFC, but the figure description in line 267-269 says "correlation between the degree of inequity and the activation of the right amygdala …".

**Response：**

Thank you for bringing this to our attention. We appreciate your feedback. In light of your observation, we have carefully reviewed and considered the structure and content of Fig3A and 3B in the new manuscript. Consequently, we have decided to merge the descriptions of Fig 3A and 3B to provide a more cohesive and streamlined presentation of the functional connectivity between the amygdala and vmPFC.

***Fig 3. Stress-induced changes in amygdala emotional salience and prefrontal executive networks in the decision phase. (A&C)*** *Stress-induced differences in brain representation of the degree of inequality and the functional connectivity between the amygdala and vmPFC.* ***(A&B)*** *PPI analysis based on the right amygdala as a seed showed that the stress group had increased functional connectivity of the right amygdala and the vmPFC during the decision stage in the punishment options. *P < 0.05; Error bars represent the SEM.* (Page 9, Line 277)

**Comment 3.4.6**
- In the mediation analyses, described in line 641-652, the authors use punishment bias as estimated from the computational model (alpha-beta) as a mediator between the effect of rACC/rPCC/rTPJ and punishment severity bias (behavioral; punishment minus help contributions). However, the model estimates are obtained directly by fitting the model to the same data that the authors are using as dependent variable (i.e., behavioral punishment severity bias). In other words, the values for the estimated punishment bias are not independently obtained. I don't think in that case the authors can run a mediation analysis.

**Response：**

Thank you for bringing up this question. We have carefully considered the major purpose and its validation of our mediation analysis. First of all, we would like to clarify that the two latent parameters of α and β in our model are estimated based on the cost of punishing and helping decisions. We have observed that α is correlated with punishment behavior, while β is correlated with help behavior. Theoretically speaking, α, β, punish, and help represent distinct psychological processes. For instance, punishing and helping behaviors may be influenced by other factors like empathy. Due to the limitations of our experimental design, we were unable to incorporate parameters related to these factors. In our current model, α represents the aversion to harming others as an allocator, while β represents the aversion to being harmed as a recipient. These two parameters are psychological components related to punish/help decisions, but they do not necessarily equate to actual demonstrated behavior. Besides, in our mediation analysis, we utilized the mediator variable, which is defined as the discrepancy between α and β (α-β). Additionally, the dependent variable we considered was the disparity between punishment and help cost (punishment-help).

In order to test that α, β, punish, and help, represent distinct psychological processes, we did some correlational analysis of the current data, taking α, β, punish, and help and correlating them separately with salivary testosterone (there is no difference between stress and control groups in salivary testosterone, see Table 3.4.6.1). We found that although the cost of help (MUs) was highly correlated with β (see Table 3.4.6.2), only the amount of help cost was negatively correlated with testosterone, whereas β was not significantly correlated with testosterone, and when controlling for β, we found that the partial correlation between the cost of help and testosterone was still significant (see Table 3.4.6.3), and thus, to some extent, the model parameters and the expected behavior do not represent exactly the same meaning. We acknowledge that further validation is necessary to establish the link between these psychological components and the expected behavior.

| **Table 3.4.6.1 Independent Samples Test of testosterone between stress and control group** | | | | | | | | |
| --- | --- | --- | --- | --- | --- | --- | --- | --- |
|  | | Levene's Test for Equality of Variances | | t-test for Equality of Means | | | | |
|  |  | F | Sig. | t | df | Sig. (2-tailed) | Mean Difference | Std. Error Difference |
| Testosterone | Equal variances assumed | 2.228 | .142 | -.453 | 50 | .653 | -.08234 | .18187 |
|  | Equal variances not assumed |  |  | -.456 | 49.606 | .651 | -.08234 | .18069 |

| **Table 3.4.6.2 Correlations between help/punish cost, α/β and testosterone** | | | | | | | | |
| --- | --- | --- | --- | --- | --- | --- | --- | --- |
|  | | β | Help cost | **α** | Punishment cost | α-β | Punish-help (cost) | Testosterone |
| β | Pearson Correlation | -- |  |  |  |  |  |  |
|  | N | 52 |  |  |  |  |  |  |
| Help cost | Pearson Correlation | .749^**^ | -- |  |  |  |  |  |
|  | Sig. (1-tailed) | .000 |  |  |  |  |  |  |
|  | N | 52 | 52 |  |  |  |  |  |
| **α** | Pearson Correlation | -.124 | -.056 | -- |  |  |  |  |
|  | Sig. (1-tailed) | .191 | .346 |  |  |  |  |  |
|  | N | 52 | 52 | 52 |  |  |  |  |
| Punishment cost | Pearson Correlation | -.103 | -.011 | .816^**^ | -- |  |  |  |
|  | Sig. (1-tailed) | .233 | .469 | .000 |  |  |  |  |
|  | N | 52 | 52 | 52 | 52 |  |  |  |
| α-β | Pearson Correlation | -.681^**^ | -.484^**^ | .811^**^ | .663^**^ | -- |  |  |
|  | Sig. (1-tailed) | .000 | .000 | .000 | .000 |  |  |  |
|  | N | 52 | 52 | 52 | 52 | 52 |  |  |
| Punish-help (cost) | Pearson Correlation | -.638^**^ | -.771^**^ | .563^**^ | .645^**^ | .792^**^ | -- |  |
|  | Sig. (1-tailed) | .000 | .000 | .000 | .000 | .000 |  |  |
|  | N | 52 | 52 | 52 | 52 | 52 | 52 |  |
| Testosterone | Pearson Correlation | -.006 | -.232^*^ | .082 | .047 | .064 | .207 | -- |
|  | Sig. (1-tailed) | .484 | .049 | .281 | .372 | .326 | .071 |  |
|  | N | 52 | 52 | 52 | 52 | 52 | 52 | 52 |
| **. Correlation is significant at the 0.01 level (1-tailed). | | | | | | | | |
| *. Correlation is significant at the 0.05 level (1-tailed). | | | | | | | | |

| **Table 3.4.6.3 Partial Correlation between help cost and testosterone** | | | | | |
| --- | --- | --- | --- | --- | --- |
| Control Variables | | | Help cost | Testosterone |  |
| β | Help cost | Correlation | 1.000 | -.343 |  |
|  |  | Significance (2-tailed) | . | .014 |  |
|  |  | df | 0 | 49 |  |
|  | Testosterone | Correlation | -.343 | 1.000 |  |
|  |  | Significance (2-tailed) | .014 | . |  |
|  |  | df | 49 | 0 |  |

Secondly, the major purpose of our mediation models is to illustrate the moderator effects of stress on relationships among these variables. We aimed to highlight the reversal of the direction of ACC and PCC concerning the punishment bias (the difference between α and β parameters) under stress, which subsequently influences actual decision making. Similar studies have employed this type of analysis to provide a more comprehensive explanation of their results, as seen in the works of(Feng et al., 2023; Zhu et al., 2021). We understand your concerns and believing that this mediation analysis may be circular due to the mediator (i.e., α vs. β) actually derived from the dependent variable (i.e., behavioral punishment severity bias). As a potential solution in response to your concern, we suggest including these results in the supplementary information (SI) to aid readers in their understanding. Considering your previous comment suggesting the removal of outliers, we have reanalyzed the data after excluding extreme values beyond three standard deviations. The current version includes the results from this reanalysis. If you prefer to include these results in the supplementary materials, we are happy to do so.

**Comment 3.4.7**
- The authors have investigated the neural correlates of the total decision utility obtained from the computational model, as well as the punishment bias (the difference between alpha and beta parameters). However, they did not try to dissect this total decision utility and study the neural correlates of the utility associated with aversion to observing someone else hurt others and aversion to observing someone else being hurt. There is no reason to assume that alpha and beta parameters (the sensitivity to inequity aversion) are represented as univariate activity of certain brain regions, rather than the three utility components comprising the total decision utility (utility to self, utility associated with aversion to observing someone else hurt others, and utility associated with aversion to observing someone else being hurt).
**Response：**

We thank you for raising very interesting comments on the three utility components comprising the total decision utility, if we understood your point correctly. We have now undertaken several steps to address the Reviewer’s concerns. First of all, we would like to clarify our original analyses which examined the neural correlates of the overall decision utility as well as punishment bias (α-β). Our computational model and recent attempts (model 5 & 6) have shown that participants did not pay more attention to weight their own interests over others’ interests. Instead, they primarily weight the fairness between options A and B, resulting in decisions to punish or help. Given the specific focus of our research, we concentrated on comparing the neural correlates associated with aversion to witnessing someone else harm others (α) and aversion to witnessing someone else being harmed (β). These aspects ultimately manifest in the neural correlates of the total decision utility associated with punishment bias (α-β). Secondly, we conducted an investigation into the neural correlates of the total decision utility associated with α and β separately. Multiple regression analyses were performed, combining each participant's α (aversion to observing someone else hurt others) and β (aversion to observing someone else being hurt) with total utility. Furthermore, we compared the differences between the stress and control groups and identified brain regions that showed conjunction. Our findings revealed that the activity of the right temporoparietal junction (rTPJ) and the right middle frontal gyrus, which correlated with parametric α, was higher in the stress group than in the control group when processing the overall utility. However, there were no significant differences observed between the stress and control groups in regions that correlated with parametric β. The findings are presented in Supplementary Material Table S9 and Table S10.

**Table S9. The neural correlates of total utility associated with aversion to witnessing someone else harm others (α).**

|  |  | **MNI Coordinates** | | | **Z score** | **voxels** |
| --- | --- | --- | --- | --- | --- | --- |
| **Brain region and contrast** | **Side** | **X** | **Y** | **Z** |  |  |
| **Control > Stress** | | | | | | |
| No region passed the threshold.  **Stress > Control** | | | | | | |
| Precuneus* | R | 16 | -56 | 46 | 5.30 | 460 |
| Angular* | R | 46 | -60 | 40 | 4.44 | 124 |
| Middle Frontal Gyrus* | R | 44 | 50 | 4 | 4.43 | 114 |
| Cerebellum^#^ | L | -38 | -74 | -44 | 4.59 | 41 |
| **Conjunction** |  |  |  |  |  |  |
| No common region found |  |  |  |  |  |  |

*Initial whole-brain threshold at P <0.001 uncorrected at voxel level and cluster corrected at P < 0.05 FWE.

^#^Initial whole-brain threshold at P _FDR_ <0.001 at voxel level and cluster corrected at P < 0.05 FWE.

**Table S10. The neural correlates of total utility associated with aversion to witnessing someone else being harmed (β).**

|  |  | **MNI Coordinates** | | | **Z score** | **voxels** |
| --- | --- | --- | --- | --- | --- | --- |
| **Brain region and contrast** | **Side** | **X** | **Y** | **Z** |  |  |
| **Stress > Control** | | | | | | |
| Precuneus | L | -26 | -56 | 52 | 3.83 | 31 |
| **Control > Stress** |  |  |  |  |  |  |
| Anterior Cingulate | L | 0 | 36 | 16 | 3.67 | 57 |
| **Conjunction**  No common region found |  |  |  |  |  |  |

Note: *The regions listed above did not pass the FWE-corrected threshold. The threshold we set* was: Initial whole-brain threshold at P <0.001 uncorrected and cluster corrected at P < 0.05 FWE.

**Comment 3.5: Discussion**
The following points all need to be addressed.

**Comment 3.5.1**
- A discussion of using CPT versus other ways of inducing acute stress in the lab and how that would change the results is missing. Existing work found the opposite results, like FeldmanHall et al 2015, should be mentioned.

**Response：**Thank you for providing this perspective. In our response to the comment #3.1, we provided some ideas related to stress induction methods. Overall, we agree that using physical methods to induce stress in a social context can reduce confusion regarding social emotions. However, considering the specific design of our experiment, which involves comparing preferences for two prosocial behaviors, we still lean towards the belief that under the induction of social stress, stress may increase the preference for helping compared to punishing. This aligns with the viewpoint presented by FeldmanHall et al. (2015), who suggested that “the increased prosocial behavior von Dawans et al. observed following the TSST may reflect an attempt to repair potentially compromised social ties by displaying greater trust”. Based on this, we hypothesize that the TSST may alter brain activity during the decision-making process related to helping. In the introduction, we cited FeldmanHall et al. (2015), Forbes et al. (2023), Schulreich et al. (2022), and Nitschke et al. (2022) to provide a comprehensive and objective description of the impact of stress on prosocial behavior.

**INTRODUCTION:**

*Decision-making under stress can follow a “tend and befriend” pattern, where decision behavior can be both adaptive and altruistic, promoting survival and well-being at both individual and group levels*(Taylor, 2006)*. Nevertheless, it is important to note that the “tend and befriend” pattern does not always hold true, as some studies have demonstrated a decrease in prosocial preferences under acute stress* (Forbes et al., 2023; Oriel FeldmanHall1 et al., 2015; Schulreich et al., 2022)*, while others have found no significant effects in economic games (according to a recent meta-analysis)* (Nitschke et al., 2022)*. Stress can profoundly impact psychological, neurophysiological, and brain networks involved in third-party decision-making, thereby influencing the affective response, motivation, and subjective value assigned to the decisions* (Phelps et al., 2014; Ulrich-Lai & Herman, 2009)*.* **(Page 2, Line 54)**

**DISCUSSION:**

*It is important to note that the sample collected for this study consisted only of males, so the results may be more applicable to men than women. Furthermore, given that a physical stressor was employed in this study, it would be intriguing to investigate the impact of a social stressor, such as the Social Stress Test (TSST), on decision preferences. We are inclined to believe that a social stressor may engage the empathy and mentalizing networks to a greater extent when making decisions related to helping. Previous research has indeed shown that social stressor can enhance prosocial behavior by intensifying the sharing of others’ emotions* (Tomova et al., 2017). **(Page 13, Line 425)**

**Comment 3.5.2**
- In the introduction, the "tend-and-befriend" hypothesis was linked to the automaticity of the helping responses, as opposed to deliberativeness of punishment; whereas in the results section the authors conclude the opposite, that punishment is an automatic response; but the behavioral results show an increase in helping behavior. And in line 359-360 states the opposite that "helping rather than punishment is the more intuitive and efficient response pattern". I wonder whether line 261-263 stated their claim in the opposite way, because the authors repeat that helping is more intuitive in line 412. Anyways, a more solid presentation of main and alternative hypotheses of this work that are based on a thorough literature review is really missing both from the introduction and from the discussion.

**Response：**

Thank you for bringing these inconsistencies to our attention. Upon careful review, we have identified that the statements in lines 261-263 were inappropriate. Based on our current research and considering previous studies, we prefer that helping is a more instinctive response compared to punishment. We have made revisions to the Introduction and Discussion sections. These revisions aim to clarify our research hypothesis and offer more cautious interpretations of the research results.

***INTRODUCTION***

*Based on the biphasic-reciprocal model of the stress-induced transition from deliberate to intuitive under stress, we hypothesized that people under stress would prefer helping when third-party punishment and helping are in conflict because helping victims is considered a more habitual response in stressful situations, which aligns with “tend and befriend” concept due to its social approach property* (Buchanan & Preston, 2014; Taylor, 2006)*. However, previous research has demonstrated that third-party punishment involves the activation of the DLPFC, which plays a crucial role in executive functioning and the integration of multiple information streams for appropriate decision-making* (Buckholtz et al., 2008; Sanfey et al., 2003; Zinchenko, 2019)*. We therefore expected that acute stress would decrease behaviors that require adequate cognitive control while increasing behaviors that recruit habitual and reflexive regulation.* (Page 3, Line 103)

*This model allowed us to estimate the parameter values reflecting individual degrees of punishment and help, with a greater punishment severity or increased help requiring more tokens to diminish the violator’s advantageous status or promote the victim’s disadvantageous status, respectively. We expected to observe neural changes in brain functioning related to the theory of mind (e.g., ACC and TPJ) and subjective value computations (e.g., vmPFC and PCC)*(Bartra et al., 2013) *under stress. This is because determining the severity of punishment or the extent of assistance is closely tied to considerations of others (such as the payoff and emotions involved) and the evaluation of available options in terms of their value.* (Page 3, Line 126)

***DISCUSSION***

*These findings suggest that when individuals are under stress, they tend to exhibit a more deliberative response pattern towards punishment rather than helping. It appears that people allocate greater cognitive resources towards value-based computation when making decisions related to punishment.* (Page 13, Line 423)

**Comment 3.5.3**
- Lines 383-385 provides an argument for the optimality of engaging in punishment decisions under stress because of the required higher cognitive resources (which is itself a reverse inference). This is an evolutionary claim, given experimental evidence for proximal mechanisms of decision making, and so it really is such a stretch given the evidence in this study, and should be stated using a more speculative language than an assertive one. Same in lines 414-415.

**Response：**

Thank you for providing these constructive suggestions, in this time, we have adopted a more open and speculative approach to some of the assertions made previously in the discussion section.

***DISCUSSION***

*Under stress, the previously observed considerate response underwent a significant transformation, and the effectiveness of punishment as a relatively desirable strategy for addressing instances of injustice involving others was diminished.* **(Page 12, Line 388)**

**Comment 3.5.4**
- I'm not sure I understand the message of the last sentence in lines 385-386.
- Line 390-392: if "previous studies demonstrated that psychological stress and early life stress increases vmPFC functional connectivity with amygdala", a finding that is not specific to a third-party intervention context, then how would the authors argue that the observed increased connectivity in their experiment is linked to decreasing punishment decisions and this is not just a "spurious" correlation? - Lines 395-397 is also stated more strongly than the presented evidence affords.
**Response：**

Thank you for providing these constructive suggestions. We have made revisions to improve clarity and logical flow in our discussion. The sentence in lines 385-386 and sentence Lines 395-397 have been deleted this time. Regarding the question about the coupling of amygdala-vmpfc, it did prompt us to think more deeply, and we have provided a brief summary of amygdala-vmpfc connectivity. In a similar study, researchers induced acute stress using SECPT to explore the neural mechanism of stress influence on self-control choices. They found that acute stress increased the influence of immediately rewarding taste, accompanied by increased functional connectivity between the ventromedial prefrontal cortex (vmPFC) and amygdala and striatal regions encoding tastiness (Maier et al., 2015). The authors suggested that this enhanced positive coupling between the vmPFC and amygdala and vStr regions may indicate the propagation of a stronger motivational signal for the tastier item into value computations. Research on third-party punishment has also found strengthened amygdala connectivity with lateral prefrontal regions involved in punishment decision-making (Treadway et al., 2014). Based on a summary of the relevant literature, amygdala-vmPFC connectivity is implicated in three broad domains of psychological function associated with psychopathology. First, it is critical for the representation of reward- and value-based decision making (Gangopadhyay et al., 2020; Maier et al., 2015). Second, it is involved in the generation and regulation of negative emotion (Wager et al., 2008). Third, it plays a role in various aspects of social cognition, such as facial emotion recognition, theory-of-mind ability, and processing self-relevant information (Hiser & Koenigs, 2018). In our own study, where we found enhanced amygdala-vmPFC coupling during punishment in the stress group, we cannot definitively determine which specific function it aligns with. As mentioned in review #3, further precise localization studies are needed. Nonetheless, we speculate that amygdala-vmPFC connectivity may potentially contribute to reward computations, regulation of aversive emotion, and social cognition during third-party punishment (Buckholtz et al., 2008). Therefore, we have adopted a more open approach to discussing these possibilities in this revision.

***DISCUSSION***

*Besides, we found that stress increased amygdala-vmPFC functional connectivity under the punishment options, previous research on third-party punishment has also discovered a strengthening of amygdala connectivity with lateral prefrontal regions that are involved in punishment decision-making*(Treadway et al., 2014) *. Amygdala-vmPFC connectivity has previously been found to play a crucial role in reward- and value-based decision-making* (Gangopadhyay et al., 2020; Maier et al., 2015)*, as well as in the generation and regulation of negative emotions* (Wager et al., 2008)*, and various aspects of social cognition, such as facial emotion recognition, theory-of-mind ability, and processing self-relevant information* (Hiser & Koenigs, 2018)*. Our findings suggest that acute stress may lead to a reduction in general activation in the amygdala’s response to inequity. However, when it comes to complex third-party punishment decision, stress may induce hypercoupling of brain networks involved in value computations, regulation of aversive emotions, and mentalizing.* **(Page 13, Line 399)**

**Comment 3.6: Other minor points**

**Comment 3.6.1**
- The FeldmanHall et al 2015 cited in line 73 is not relevant to the claim the authors are making "[ToM brain regions which are] fundamental for becoming more other-oriented with a preference for providing help".

**Response：**

Thank you for bringing this to our attention. After reviewing the citation for the content, we have confirmed that it should reference the study conducted by Tomova et al. in 2017. We have made the appropriate changes to the manuscript to reflect this correction.

***INTRODUCTION***

Additionally, acute stress has been found to increase activation in brain regions associated with Theory of Mind (ToM), which plays a crucial role in altruistic behavior(Tomova et al., 2017; Tusche et al., 2016). (Page 2, Line 72)

**Comment 3.6.2**
- The authors wrote "during CPT, the stress group exhibited higher levels of salivary cortisol". However, Fig 1B suggests that cortisol is only significantly different in the third-party intervention phase and not in the stress induction phase.

**Response：**

We would like to provide clarification regarding the term “stress induction phase” mentioned in the previous manuscript. In our study, the “stress induction phase” refers to the 3-minute period of the Cold Pressor Test (CPT) task. This phase is characterized by a relatively slow response of the hypothalamic-pituitary-adrenal (HPA) axis, and the secretion of cortisol has not yet reached a significant higher level. Our findings align with numerous classic studies, such as the research conducted by FeldmanHall et al. in 2015. In their study, the stress group exhibited a substantial increase in cortisol levels only within the 10–40-minute timeframe following the CPT task.

**Comment 3.6.3**
- There are two p-values reported for the group x time interaction for salivary cortisol level result on page 4.

**Response：**

Thank you for bringing this mistake to our attention. We have made the corrections in the revised manuscript.

***RESULTS***

*This analysis revealed a significant Group × Time interaction for salivary cortisol level (F (2.22, 110.75) = 11.231, P < 0.001, η_p_^2^ = 0.183), self-reported negative affect (F(2, 98) = 7.38, P < 0.001, η_p_^2^ = 0.131) and heart rate (F (1, 49) = 18.19, P < 0.001, η_p_^2^ = 0.271).* (Page4, Line149)

**Comment 3.6.4**
- Fig 1E: spelling error in deduct written as "decuct"

**Response：**

Thank you for bringing this mistake to our attention. We have made the spelling correction in the Fig 1E.

**Comment 3.6.5**
- Fig 2A: the x-axis and the significance signs are not aligned with the bars. Also, it would be better to use "keep" instead of "self" for the x-axis labels for more consistency.

**Response：**

The significance signs and x-axis labels in Fig 2A have been modified according to your suggestions.

**Comment 3.6.6**
- What are the small patterned squares in Fig 2D?

**Response：**

The small patterned squares in Fig 2D depict the predicted cost of punishment and help as determined by our win model. To facilitate easy comparison, we have included both the actual cost and the predicted cost on the same graph.

**Comment 3.6.7**
- There is no scatter plot in Fig 2B&D, as mentioned in line 210-211.

**Response：**

The statement in lines 210-211, “The scatter plot depicts the correlation between the actual and predicted contributions based on computational modeling,” was initially meant to describe the relationship between the predicted cost of punishment and help as determined by our win model (small patterned squares displayed in Fig 2D) and the actual cost. However, upon further consideration, we recognized that this specific information is solely presented in Fig 2D, and the phrase “correlation between the actual and predicted contributions” may not accurately capture the intended meaning. Finally, we have revised the legends of both Fig 2B and Fig 2D to provide separate explanations for each.

**Comment 3.6.8**
- Fig 3, line 275, D-G not D&G for referring to a range. Same for E&G.

**Response：**

Thank you for bringing this mistake to our attention. We have made the correction in Fig 3.

**Comment 3.6.9**
- Line 337, "through acting emotional salience" was perhaps intended to be "acting upon" or "activating" or "affecting"?

**Response：**

Thank you for your suggestion. We appreciate your input, and we have taken it into consideration in the revised version. We agree that the phrase “acting upon” better captures the intended meaning, and we have made the amendment in the text.

***DISCUSSION***

*These findings will be discussed in a framework of behavioral and neurocomputational mechanisms of how acute stress affects third parties’ decisions acting upon emotional salience, prefrontal control and mentalizing networks.* (Page 11, Line 348)

**Comment 3.6.10**
- "correspo" spelling error in line 389

**Response：**

Thank you for bringing this mistake to our attention. We have made the spelling correction in the manuscript.

***DISCUSSION***

*In conjunction with local activation, we found that stress increased amygdala-vmPFC functional connectivity, and this connectivity, in turn, inhibited the punishment option, corresponds to the early findings that the degree of inequity aversion is predictable from amygdala activity*(Haruno & Frith, 2010)*.* (Page 12, Line 392)

**Comment 3.6.11**
- Line 466, "how many" is written as "how any"

**Response：**

Thank you for bringing this mistake to our attention. We have made the spelling correction in the manuscript.

***MATERIALS AND METHODS***

*In the decision stage, participants were presented with three options: transferring MUs to decrease player A’s MUs, transferring MUs to increase player B’s MUs, or keeping all the MUs for themselves. If participants chose to reduce player A’s money during the decision stage, they could first decide on the amount of MUs to transfer from their own initial 50 MUs and deduct that amount from player A’s MUs in increments of 5 MUs in the first transfer stage. Following that, participants could further decide on the amount of MUs to transfer from their remaining MUs to compensate player B in the second transfer stage.* (Page 14, Line 477)

**Comment 3.6.12**
- Line 503, grammar error in "we fitted behavioral data to computational modeling"

**Response：**

Thank you for bringing this mistake to our attention. We have made the corrections in the manuscript.

***MATERIALS AND METHODS***

***Computational modeling procedures.*** To better understand the neural *mechanisms* underlying *acute stress and its impact on third-party intervention behaviors, we employed computational modeling to analyze the behavioral data. This approach enabled us to evaluate the extent to which unrelated third parties were personally invested in addressing inequality aversion.* (Page 15, Line 526)

**Comment 3.6.13**
- "citation" in line 525 is missing

**Response：**

Thank you for bringing this mistake to our attention. We have made the corrections in the manuscript.

***MATERIALS AND METHODS***

***Model comparisons.*** *To assess whether the “other-regarding inequality aversion model” provided a compelling explanation of participants’ behavior, here, three alternative models were compared by calculating the Akaike Information Criterion (AIC) for each model*(Stallen et al., 2018)*.* (Page 16, Line 551)

**Comment 3.6.14**
- be more explicit about the definition of height and extent threshold in line 227.
**Response：**

Thank you for your suggestion. In this revised version, we have made consistent changes throughout the entire manuscript regarding the threshold in relation to MRI analysis. Specifically, we have clarified that the “height” refers to the voxel level threshold (*P* _uncorrected_ < 0.001), while the “extent” refers to the cluster level threshold (*P* _FWE_ < 0.05). This modification ensures clear and precise terminology, helping readers understand the thresholds and their respective levels more accurately.

**References**

Arnsten, A. F. T. (2015). Stress weakens prefrontal networks: molecular insults to higher cognition. *Nature Neuroscience*, *18*(10), 1376–1385. https://doi.org/10.1038/nn.4087

Bartra, O., McGuire, J. T., & Kable, J. W. (2013). The valuation system: A coordinate-based meta-analysis of BOLD fMRI experiments examining neural correlates of subjective value. *NeuroImage*, *76*, 412–427. https://doi.org/10.1016/j.neuroimage.2013.02.063

Bellucci, G., Camilleri, J. A., Eickhoff, S. B., & Krueger, F. (2020). Neural signatures of prosocial behaviors. *Neuroscience and Biobehavioral Reviews*, *118*(May), 186–195. https://doi.org/10.1016/j.neubiorev.2020.07.006

Botvinick, M. M. (2007). Conflict monitoring and decision making: reconciling two perspectives on anterior cingulate function. *Cognitive, Affective & Behavioral Neuroscience*, *7*(4), 356–366. https://doi.org/10.3758/CABN.7.4.356

Boyd, R., Gintis, H., & Bowles, S. (2010). Coordinated punishment of defectors sustains cooperation and can proliferate when rare. *Science*, *328*(5978), 617–620. https://doi.org/10.1126/SCIENCE.1183665/SUPPL_FILE/BOYD-SOM.PDF

Boyd, R., Gintis, H., Bowles, S., & Richerson, P. J. (2003). The evolution of altruistic punishment. *Proceedings of the National Academy of Sciences of the United States of America*, *100*(6), 3531–3535. https://doi.org/10.1073/pnas.0630443100

Buchanan, T. W., & Preston, S. D. (2014). Stress leads to prosocial action in immediate need situations. In *Frontiers in Behavioral Neuroscience* (Vol. 8, Issue JAN). https://doi.org/10.3389/fnbeh.2014.00005

Büchel, C., Holmes, A. P., Rees, G., & Friston, K. J. (1998). Characterizing stimulus-response functions using nonlinear regressors in parametric fMRI experiments. *NeuroImage*. https://doi.org/10.1006/nimg.1998.0351

Buckholtz, J. W., Asplund, C. L., Dux, P. E., Zald, D. H., Gore, J. C., Jones, O. D., & Marois, R. (2008). The Neural Correlates of Third-Party Punishment. *Neuron*, *60*(5), 930–940. https://doi.org/10.1016/j.neuron.2008.10.016

Burghy, C. a, Stodola, D. E., Ruttle, P. L., Molloy, E. K., Jeffrey, M., Oler, J. a, Fox, M. E., Hayes, A. S., Kalin, N. H., Essex, M. J., Davidson, R. J., & Birn, R. M. (2013). Developmental pathways to amygdala-prefrontal function and internalizing symptoms in adolescence. *Nature Neuroscience*, *15*(12), 1736–1741. https://doi.org/10.1038/nn.3257.Developmental

Carmichael, O., & Lockhart, S. (2018). Neurobiological programming of early life stress: Functional development of amygdala-prefrontal circuitry and vulnerability for stress-related psychopathology. *Curr Top Behav Neurosci*, *November 2011*, 289–320. https://doi.org/10.1007/7854

Civai, C., Crescentini, C., Rustichini, A., & Rumiati, R. I. (2012). Equality versus self-interest in the brain: Differential roles of anterior insula and medial prefrontal cortex. *NeuroImage*, *62*(1), 102–112. https://doi.org/10.1016/J.NEUROIMAGE.2012.04.037

Civai, C., Huijsmans, I., & Sanfey, A. G. (2019). Neurocognitive mechanisms of reactions to second- and third-party justice violations. *Scientific Reports*, *9*(1), 1–11. https://doi.org/10.1038/s41598-019-45725-8

Fehr, E., & Fischbacher, U. (2004). Third-party punishment and social norms. *Evolution and Human Behavior*, *25*(2), 63–87. https://doi.org/10.1016/S1090-5138(04)00005-4

Fehr, E., Ga, S., & Gächter, S. (2002). Altruistic punishment in humans. *NATURE*, *415*(6868), 137–140. https://doi.org/10.1038/415137a

FeldmanHall, O., Sokol-Hessner, P., Van Bavel, J. J., & Phelps, E. A. (2014). Fairness violations elicit greater punishment on behalf of another than for oneself. *Nature Communications*, *5*, 1–6. https://doi.org/10.1038/ncomms6306

Feng, C., Luo, Y. J., & Krueger, F. (2015). Neural signatures of fairness-related normative decision making in the ultimatum game: A coordinate-based meta-analysis. *Human Brain Mapping*, *36*(2), 591–602. https://doi.org/10.1002/hbm.22649

Feng, C., Tian, X., & Luo, Y.-J. (2023). Neurocomputational substrates underlying the effect of identifiability on third-party punishment. *The Journal of Neuroscience*, JN-RM-0460-23. https://doi.org/10.1523/JNEUROSCI.0460-23.2023

Forbes, P. A. G., Aydogan, G., Braunstein, J., Todorova, B., Wagner, I. C., Lockwood, P. L., Apps, M. A. J., Ruff, C. C., & Lamm, C. (2023). Acute stress reduces effortful prosocial behaviour. *ELife*, *12*. https://doi.org/10.7554/ELIFE.87271

Gangopadhyay, P., Chawla, M., Dal Monte, O., & Chang, S. W. C. (2020). Prefrontal–amygdala circuits in social decision-making. *Nature Neuroscience 2020 24:1*, *24*(1), 5–18. https://doi.org/10.1038/s41593-020-00738-9

Gao, X., Yu, H., Sáez, I., Blue, P. R., Zhu, L., Hsu, M., & Zhou, X. (2018). Distinguishing neural correlates of context-dependent advantageous- and disadvantageous-inequity aversion. *Proceedings of the National Academy of Sciences of the United States of America*, *115*(33), E7680–E7689. https://doi.org/10.1073/pnas.1802523115

Ginty, A. T., Kraynak, T. E., Kuan, D. C., & Gianaros, P. J. (2019). Ventromedial prefrontal cortex connectivity during and after psychological stress in women. *Psychophysiology*, *56*(11), 1–15. https://doi.org/10.1111/psyp.13445

Haruno, M., & Frith, C. D. (2009). Activity in the amygdala elicited by unfair divisions predicts social value orientation. *Nature Neuroscience 2009 13:2*, *13*(2), 160–161. https://doi.org/10.1038/nn.2468

Haruno, M., & Frith, C. D. (2010). Activity in the amygdala elicited by unfair divisions predicts social value orientation. *Nature Neuroscience*, *13*(2), 160–161. https://doi.org/10.1038/nn.2468

Hermans, E. J., Henckens, M. J. A. G., Joels, M., Fernandez, G., Joëls, M., & Fernández, G. (2014). Dynamic adaptation of large-scale brain networks in response to acute stressors. *Trends in Neurosciences*, *37*(6), 304–314. https://doi.org/10.1016/j.tins.2014.03.006

Hiser, J., & Koenigs, M. (2018). The Multifaceted Role of the Ventromedial Prefrontal Cortex in Emotion, Decision Making, Social Cognition, and Psychopathology. *Biological Psychiatry*, *83*(8), 638–647. https://doi.org/10.1016/J.BIOPSYCH.2017.10.030

Hu, Y., Strang, S., & Weber, B. (2015). Helping or punishing strangers: Neural correlates of altruistic decisions as third-party and of its relation to empathic concern. *Frontiers in Behavioral Neuroscience*, *9*(FEB), 24. https://doi.org/10.3389/fnbeh.2015.00024

Krueger, F., & Hoffman, M. (2016). The Emerging Neuroscience of Third-Party Punishment. *Trends in Neurosciences*, *39*(8), 499–501. https://doi.org/10.1016/j.tins.2016.06.004

Li, Y., Hu, J., Ruff, C. C., & Zhou, X. (2022). *Neurocomputational evidence that conflicting prosocial motives guide distributive justice*. https://doi.org/10.1073/pnas.2209078119

Maier, S. U. U. U., Makwana, A. B. B. B., & Hare, T. A. A. A. (2015). Acute Stress Impairs Self-Control in Goal-Directed Choice by Altering Multiple Functional Connections within the Brain’s Decision Circuits. *Neuron*, *87*(3), 621–631. https://doi.org/10.1016/j.neuron.2015.07.005

Nickels, N., Kubicki, K., & Maestripieri, D. (2017). Sex Differences in the Effects of Psychosocial Stress on Cooperative and Prosocial Behavior: Evidence for ‘Flight or Fight’ in Males and ‘Tend and Befriend’ in Females. *Adaptive Human Behavior and Physiology*, *3*(2), 171–183. https://doi.org/10.1007/s40750-017-0062-3

Nitschke, J. P., Forbes, P. A. G., & Lamm, C. (2022). Does stress make us more—or less—prosocial? A systematic review and meta-analysis of the effects of acute stress on prosocial behaviours using economic games. *Neuroscience & Biobehavioral Reviews*, *142*, 104905. https://doi.org/10.1016/J.NEUBIOREV.2022.104905

Oosterbeek, H., Sloof, R., & Van De Kuilen, G. (2004). Cultural differences in ultimatum game experiments: Evidence from a meta-analysis. *Experimental Economics*. https://doi.org/10.1023/B:EXEC.0000026978.14316.74

Oriel FeldmanHall1, C. M. R., FeldmanHall, O., Raio, C. M., Kubota, J. T., Seiler, M. G., & Phelps, E. A. (2015). The Effects of Social Context and Acute Stress on Decision Making Under Uncertainty. *Psychological Science*, *26*(12), 1918–1926. https://doi.org/10.1177/0956797615605807

Phelps, E. A., & LeDoux, J. E. (2005). Contributions of the amygdala to emotion processing: From animal models to human behavior. In *Neuron* (Vol. 48, Issue 2). https://doi.org/10.1016/j.neuron.2005.09.025

Phelps, E. A., Lempert, K. M., & Sokol-Hessner, P. (2014). Emotion and Decision Making: Multiple Modulatory Neural Circuits. *Annual Review of Neuroscience*, *37*(1), 263–287. https://doi.org/10.1146/annurev-neuro-071013-014119

Sanfey, A. G., Rilling, J. K., Aronson, J. A., Nystrom, L. E., & Cohen, J. D. (2003). The neural basis of economic decision-making in the Ultimatum Game. *Science*, *300*(5626), 1755–1758. https://doi.org/10.1126/science.1082976

Schulreich, S., Tusche, A., Kanske, P., & Schwabe, L. (2022). Altruism under Stress: Cortisol Negatively Predicts Charitable Giving and Neural Value Representations Depending on Mentalizing Capacity. *Journal of Neuroscience*, *42*(16), 3445–3460. https://doi.org/10.1523/JNEUROSCI.1870-21.2022

Sinha, R., Lacadie, C. M., Todd Constable, R., & Seo, D. (2016). Dynamic neural activity during stress signals resilient coping. *Proceedings of the National Academy of Sciences of the United States of America*, *113*(31), 8837–8842. https://doi.org/10.1073/PNAS.1600965113

Stallen, M., Rossi, F., Heijne, A., Smidts, A., De Dreu, C. K. W. W., & Sanfey, A. G. (2018). Neurobiological Mechanisms of Responding to Injustice. *The Journal of Neuroscience*, 1242–17. https://doi.org/10.1523/JNEUROSCI.1242-17.2018

Strobel, A., Zimmermann, J., Schmitz, A., Reuter, M., Lis, S., Windmann, S., & Kirsch, P. (2011). Beyond revenge: neural and genetic bases of altruistic punishment. *NeuroImage*, *54*(1), 671–680. https://doi.org/10.1016/j.neuroimage.2010.07.051

Takahashi, T., Ikeda, K., & Hasegawa, T. (2007). Social evaluation-induced amylase elevation and economic decision-making in the dictator game in humans. *Neuroendocrinology Letters*, *28*(5), 662–665.

Taylor, S. E. (2006). Tend and befriend: Biobehavioral bases of affiliation under stress. *Current Directions in Psychological Science*, *15*(6), 273–277. https://doi.org/10.1111/j.1467-8721.2006.00451.x

Tomova, L., Majdandžić, J., Hummer, A., Windischberger, C., Heinrichs, M., & Lamm, C. (2017). Increased neural responses to empathy for pain might explain how acute stress increases prosociality. *Social Cognitive and Affective Neuroscience*, *12*(3), 401–408. https://doi.org/10.1093/scan/nsw146

Treadway, M. T., Buckholtz, J. W., Martin, J. W., Jan, K., Asplund, C. L., Ginther, M. R., Jones, O. D., & Marois, R. R. R. (2014). Corticolimbic gating of emotion-driven punishment. *Nature Neuroscience*, *17*(9), 1270–1275. https://doi.org/10.1038/nn.3781

Tusche, A., Böckler, A., Kanske, P., Trautwein, F. M., & Singer, T. (2016). Decoding the Charitable Brain: Empathy, Perspective Taking, and Attention Shifts Differentially Predict Altruistic Giving. *The Journal of Neuroscience : The Official Journal of the Society for Neuroscience*, *36*(17), 4719–4732. https://doi.org/10.1523/JNEUROSCI.3392-15.2016

Tusche, A., & Hutcherson, C. A. (2018). Cognitive regulation alters social and dietary choice by changing attribute representations in domain-general and domain-specific brain circuits. *ELife*, *7*. https://doi.org/10.7554/ELIFE.31185

Tzourio-Mazoyer, N., Landeau, B., Papathanassiou, D., Crivello, F., Etard, O., Delcroix, N., Mazoyer, B., & Joliot, M. (2002). Automated anatomical labeling of activations in SPM using a macroscopic anatomical parcellation of the MNI MRI single-subject brain. *NeuroImage*. https://doi.org/10.1006/nimg.2001.0978

Ulrich-Lai, Y. M., & Herman, J. P. (2009). Neural regulation of endocrine and autonomic stress responses. *Nature Reviews Neuroscience*, *10*(6), 397–409. https://doi.org/10.1038/nrn2647

von Dawans, B., Fischbacher, U., Kirschbaum, C., Fehr, E., & Heinrichs, M. (2012). The Social Dimension of Stress Reactivity. *Psychological Science*, *23*(6), 651–660. https://doi.org/10.1177/0956797611431576

Wager, T. D., Davidson, M. L., Hughes, B. L., Lindquist, M. A., & Ochsner, K. N. (2008). Prefrontal-Subcortical Pathways Mediating Successful Emotion Regulation. *Neuron*, *59*(6), 1037–1050. https://doi.org/10.1016/J.NEURON.2008.09.006

Xie, E., Liu, M., Liu, J., Gao, X., & Li, X. (2022). Neural mechanisms of the mood effects on third-party responses to injustice after unfair experiences. *Human Brain Mapping*. https://doi.org/10.1002/hbm.25874

Yu, R., Calder, A. J., & Mobbs, D. (2014). Overlapping and distinct representations of advantageous and disadvantageous inequality. *Human Brain Mapping*, *35*(7), 3290–3301. https://doi.org/10.1002/HBM.22402

Zhu, R., Xu, Z., Su, S., Feng, C., Luo, Y., Tang, H., Zhang, S., Wu, X., Mai, X., & Liu, C. (2021). From gratitude to injustice: Neurocomputational mechanisms of gratitude-induced injustice. *NeuroImage*, *245*. https://doi.org/10.1016/j.neuroimage.2021.118730

Zinchenko, O. (2019). Brain responses to social punishment: a meta-analysis. *Scientific Reports*, *9*(1). https://doi.org/10.1038/s41598-019-49239-1
